# Development of a single-chain variable antibody fragment against a conserved region of the SARS-CoV-2 spike protein

**DOI:** 10.1038/s41598-024-64103-7

**Published:** 2024-06-22

**Authors:** Tingyu Gao, Atsushi Irie, Takahisa Kouwaki, Hiroyuki Oshiumi

**Affiliations:** Department of Immunology, Graduate School of Medical Sciences, Faculty of Life Sciences, Kumamoto University, 1-1-1, Honjo, Chuo-ku, Kumamoto, 860-8556 Japan

**Keywords:** SARS-CoV-2, Protein design, Antibody generation, Antibody isolation and purification

## Abstract

Severe acute respiratory syndrome coronavirus 2 (SARS-CoV-2) has prolonged the duration of the pandemic because of the continuous emergence of new variant strains. The emergence of these mutant strains makes it difficult to detect the virus with the existing antibodies; thus, the development of novel antibodies that can target both the variants as well as the original strain is necessary. In this study, we generated a high-affinity monoclonal antibody (5G2) against the highly conserved region of the SARS-CoV-2 spike protein to detect the protein variants. Moreover, we generated its single-chain variable antibody fragment (sc5G2). The sc5G2 expressed in mammalian and bacterial cells detected the spike protein of the original SARS-CoV-2 and variant strains. The resulting sc5G2 will be a useful tool to detect the original SARS-CoV-2 and variant strains.

## Introduction

The 2019 coronavirus pandemic, which was caused by SARS-CoV-2, continues to pose an unprecedented threat to public health^1^. Vaccination has proven to be effective in disease prevention and control^2^; however, the continued global spread of SARS-CoV-2 has resulted in the emergence of many new variant strains^3,4^. Mutations in the spike protein can alter the antigenic identity, resulting in missed diagnosis and failure of vaccine-induced immune protection^5–7^.

The SARS-CoV-2 spike protein plays a pivotal role in the pathogenesis of COVID-19 and is a major target of vaccine development^8–10^. The spike protein forms a homotrimer on the virus surface that is cleaved into two fragments, S1 and S2, after the virus binds to angiotensin-converting enzyme 2 (ACE2), its cell surface target^11,12^. The S1 fragment contains the ACE2 binding domain and many amino acid substitutions in the S1 fragment have been detected in the variants. In contrast, the S2 fragment mediates cell entry through a fusion process between the viral and host cell membranes. Thus, the S2 region is relatively conserved among variants compared with the S1 region^13,14^.

In this study, we focused on the conserved region of the spike protein to develop an antibody that can detect any SARS-CoV-2 variants. We produced a monoclonal antibody (5G2) against the conserved S2 region of the SARS-CoV-2 spike protein. Then, we generated a single-chain form of the antibody variable regions of the heavy and light chains (sc5G2) for easier production and modification of its function by protein engineering and chemical modification. The sc5G2 was fused with a 6xHis-tag (sc5G2-6xHis) and with the maltose-binding protein (MBP) to facilitate purification and detection (MBP-sc5G2-6xHis). The vectors carrying the MBP-sc5G2-6xHis gene efficiently induced relatively high expression of the recombinant antibody in mammalian HEK293FT and *E. coli* cells and the vectors carrying the sc5G2-6xHis gene were, to a lesser extent, also expressed in *E. coli* cells. These modified antibodies reacted with spike proteins of the WT (Wuhan strain) and α, β, γ, and ο (BQ1.1, BA.5)^15,16^ variants of SARS-CoV-2. The data indicate that these antibodies could target variants of SARS-CoV-2 that are free from mutations in the epitope region and will provide a useful platform to deliver drugs or molecules that prevent many variants of SARS-CoV-2 infection.

## Materials and methods

### Cells and reagents

HEK293FT and VeroE6/TMPRSS2 (Vero)^17,18^ cells were cultured in Dulbecco’s Modified Eagle’s Medium (DMEM) with 10% fetal bovine serum (FBS), 2 mM L-glutamine solution and MEM non-essential amino acids solution (100 × diluted) (FUJIFILM, Tokyo, Japan), 100 U/mL penicillin, and 100 μg/mL streptomycin (Nacalai Tesque, Tokyo, Japan) in a humidified incubator containing 5% CO_2_ at 37 °C. The SARS-CoV-2 spike 1099–1112 peptide (GTH peptide: GTHWFVTQRNFYEP) was synthesized by Synpeptide Co., Ltd. (Shanghai, China). Anti-MBP tag mouse monoclonal antibody (mAb), anti-6xHis-Tag mouse mAb (27E8), and anti-mouse IgG-F(ab’)_2_-HRP were purchased from Proteintech, Cell Signaling Technology, and Cytiva, respectively. The 6xHis-tagged recombinant SARS-CoV-2 (2019-nCoV) spike proteins of S1 + S2, S1 and S2 extracellular domains and the codon-optimized full-length SARS-CoV-2 spike gene were obtained from Sino Biological.

### Immunization of mice

Blood was collected from the mice before immunization with the GTH peptide. HLA-DR4 transgenic mice^19^ were immunized three times with 100 µg of peptide and Freund’s adjuvant (Sigma-Aldrich). For the first immunization, complete Freund's adjuvant was used and for the second and third immunizations, incomplete Freund's adjuvant was used. The interval between immunizations was one week. Seven days after the last immunization, blood was collected, and sera was prepared by leaving the blood at RT overnight and stored at 4 ℃ until use.

### Enzyme-linked immunosorbent assay (ELISA)

ELISA was performed in 96-well plates (MaxiSorp; Thermo Fisher). Each well was coated with 50 μL of the GTH peptide (40 ng/mL) or virus (1 × 10^5^) in PBS, and the plates were stored at 4 ℃ overnight. Blocking buffer (0.05% Tween20 and 3% BSA in PBS) was then added to each well. Anti-serum diluted with PBS-T (0.05% Tween 20 in PBS) was added to each well, the plates were incubated for 1 h at RT, and washed four times with PBS-T. Anti-mouse IgG-F(ab’)_2_-HRP diluted in PBS-T was added to the wells and incubated for 1 h at RT. After washing the plates four times with PBS-T, 100 μL of the TMB solution (Invitrogen) was added to each well. The reaction was terminated by adding 50 μL of 2 N H_2_SO_4_ and the absorbance was measured at 450 nm. The statistical significance was determined by one-way analysis of variance (one-way ANOVA). All data were expressed as the mean ± standard error (SE) of the mean. Probability values (*P* values) of 0.05 or less were considered significant. All experiments were independently repeated two or more times to confirm reproducibility.

### Production of the mAbs

mAbs against the GTH peptide were produced at Medical Chemistry Pharmaceutical Co., LTD. (Hokkaido, Japan) by immunizing GANP mice with the peptide^20,21^. The hybridoma supernatants were screened by ELISA as described above. The mAb was purified using a HiTrap Protein G column (GE Healthcare) according to the manufacturer's instructions. The dissociation constant (*K*_d_) value of the purified mAb was determined by surface plasmon resonance at FUJIFILM Wako Pure Chemical Corporation (Tokyo, Japan).

### Production of recombinant anti-spike antibody

Total RNA from the anti-GTH peptide hybridoma designated 5G2, which showed the highest affinity to the peptide by ELISA, was isolated using the RNeasy kit (Qiagen) according to the manufacturer's instructions. Complementary DNA (cDNA) was synthesized using the SMARTScribe RT system (Clontech) and the immunoglobulin heavy and light chain cDNAs were amplified using a method described by Mayer et al.^22^. The PCR products were TA-cloned and sequenced using an Applied Biosystems 3130 Genetic Analyzer.

The V(D)J fragment, junction details, and N nucleotide insertions were analyzed using the NCBI IgBLAST site (https://www.ncbi.nlm.nih.gov/igblast/). The heavy and light chain variable region cDNAs were connected to the cDNAs of the mouse IgG constant γ1 and κ chains, respectively, and the full-length cDNAs were ligated into the *XhoI/XbaI* sites of the pHEK293 Ultra Expression Vector II (pHEK-II vector, Takara Bio Inc). The heavy and light chain expression vectors were co-transfected into HEK293FT cells using Lipofectamine 2000 (LF2000, Invitrogen) based on the manufacturer's instructions^23^. After 4–5 days, the culture supernatant was collected and 1 mL of the aliquot was used for the western blot as described below to confirm the reactivity to the recombinant spike proteins (Sino Biological).

### Construction of single-chain recombinant 5G2 expression vectors

The cDNAs from the variable regions from the heavy and the light chains were linked by a linker (GGTGGAGGCGGTTCAGGCGGAGGTGGCTCTGGCGGTGGCGGATCT) and the cDNA was ligated into the *NcoI/XhoI* sites of the pET22b vector (Novagen) to generate pET22b/sc5G2-6xHis for bacterial cell expression. In addition, the sc5G2 sequence followed by a 6xHis coding sequence (CACCACCACCACCACCACTGA) was ligated into the *EcoRI/XbaI* sites of the pMAL-p2 vector (New England Biolabs). The cDNA encoding MBP-sc5G2-6xHis was amplified and ligated into the pHEK-II or pET22b vectors to generate pHEK-II/MBP-sc5G2-6xHis or pET22b/MBP-sc5G2-6xHis for mammalian and bacterial cell expression, respectively^24^.

### Mammalian cell expression of MBP-sc5G2-6xHis and purification

The pHEK-II/MBP-sc5G2-6xHis was transfected into 3.0 × 10^7^ HEK293FT cells in five 10 cm dishes using LF2000 (Invitrogen) reagent based on the manufacturer's instructions. The cells were cultured in 1% FBS DMEM and the medium was recovered every other day for a total of 5 times. The recovered media were combined, and imidazole (FUJIFILM) was added to a final concentration of 20 mM. The solution was centrifuged at 20,000 × g for 20 min and filtered (0.45 μm). The filtered medium was applied to a HisTrap 5 mL column (GE Healthcare). After washing with 25 mL of HisTrap binding buffer (20 mM sodium phosphate, 0.5 M NaCl, 20 mM imidazole, pH 7.4), the bound proteins were eluted with the HisTrap elution buffer (20 mM sodium phosphate, 0.5 M NaCl, 200 mM imidazole, pH 7.4). Aliquots of 5 mL were collected. Fractions #2 and #3 were combined and applied to an MBPTrap 1 mL column (GE Healthcare). After washing the column with 5 mL of MBPTrap binding buffer (20 mM Tris–HCl, 200 mM NaCl, 1 mM EDTA, pH 7.4), the MBP-sc5G2-6xHis was eluted with MBPTrap elution buffer (10 mM maltose in MBPTrap binding buffer). Aliquots of 1 mL were collected and 10 μL of #2 fraction was subjected to SDS-PAGE. The purity was verified by Coomassie Brilliant Blue (CBB) staining and by western blot using anti-MBP antibody and anti-mouse IgG-F(ab’)_2_-HRP, respectively.

### Bacterial cell expression and purification of recombinant proteins

*E. coli* BL21(DE3)^25,26^ was transformed with pET22b/MBP-sc5G2-6xHis or pET22b/sc5G2-6xHis and cultured in 100 mL of LB media at 37 °C to an A_600_ of approximately 0.5. IPTG was added to a final concentration of 0.3 mM and cultured for 6 h at RT. Cells were harvested by centrifugation at 7,000 × g for 10 min at 4 °C and the supernatant was discarded. The pellet was resuspended in 40 mL of 30 mM Tris pH 8.0, 20% sucrose, 1 mM EDTA, and incubated for 10 min at RT with shaking. The cells were then centrifuged at 10,000 × *g* for 10 min at 4 °C and the pellet was resuspended in 5 mM Mg_2_SO_4_ and incubated for 10 min on ice with shaking. The cells were centrifuged at 10,000 × *g* for 10 min at 4 °C and the pellet was recovered^27^.

The pellet was suspended in 5 mL of 50 mM Tris, 200 mM NaCl, pH 8.0, and subjected to seven freeze–thaw cycles. After shearing by passing through an 18 G needle, the suspension was centrifuged at 5800 × *g* for 30 min at 4 °C. The pellet was resuspended in 6 M guanidine hydrochloride (GdnHCl) and 5 mM imidazole in TBS (50 mM Tris–HCl, 200 mM NaCl, pH 8.0) and stored overnight at 4 °C. The solution was centrifuged at 5,800 g for 30 min at 4 °C and the supernatant was filtered (0.45 μm) and applied to a HisTrap 5 mL column equilibrated with the same buffer. After washing the column with the buffer, the MBP-sc5G2-6xHis was eluted with 6 M GdnHCl and 200 mM imidazole in TBS. Next, 2-mercaptoethanol (167μL) was added to the aliquot #2 and stored overnight at 4 °C. The A_280_ was measured and the protein concentration was adjusted to 20 μM with 6 M GdnHCl in TBSE buffer (50 mM Tris–HCl, pH 8.0, 200 mM NaCl, 1 mM EDTA). Two mL of the solution were successively dialyzed for 12 h at 4 °C with 100 mL each of 6 M, 3 M, and 2 M GdnHCl in TBSE. The protein solution was then dialyzed for 12 h at 4 °C against 100 mL of 1 M GdnHCl, 0.4 M arginine hydrochloride and 20 mM glutathione (reduced: oxidized = 9:1) in TBSE, and 0.5 M GdnHCl in TBSE. Finally, GdnHCl was removed by the dialysis three times with 100 mL of TBSE at 4 °C for 6 h.

The refolded MBP-sc5G2-6xHis was subjected to the MBPTrap column (1 mL) and further purified as described in the "Mammalian cell expression of MBP-sc5G2-6xHis and purification" section. The aliquot #2 was subjected to SDS-PAGE and the purity was verified by CBB staining and by western blot using anti-MBP antibody and anti-mouse IgG-F(ab’)_2_-HRP.

To produce recombinant S2 without 6xHis, a cDNA encoding the S2 domain of the codon-optimized spike was amplified using the following primers: CCGGCGATGGCCATGGCTCTGATTGCCAACCAGTTC and TGGTTGGCAATCAGGCCTCCTGATGATCCGCCCAGTTCTTGGAGGTCAA. The PCR product was ligated into a TA vector (pMD20T, TAKARA Bio Inc., Shiga, Japan). The plasmid was then digested with *SpeI*, treated with Klenow fragment, and self-ligated to introduce a stop codon. The S2 cDNA was excised by *NcoI/HindIII* digestion and ligated into the pET22b vector for *E. coli* expression as described above.

### Preparation of SARS-CoV-2 viruses for ELISA

The procedure was entirely done in a P3 laboratory. Vero cells were cultured using 1% FBS in DMEM. Vero cells were infected with SARS-CoV-2 strains (WT, α, β, r and o) for 24 h. The supernatant was centrifuged at 2,000 rpm for 10 min and dispensed at 200 μL/tube. The viruses were titrated by plaque assay and stored at − 80 °C. Before use, the viruses were inactivated at 56 °C for 30 min.

### Vero cell infection with SARS-CoV-2 viruses for western blot

Vero cells (Vero E6/TMPRESS2) (1 × 10^6^) were added to a 6-well plate with 2 mL of 1% FBS in DMEM and incubated overnight. Each SARS-CoV-2 strain (2 × 10^3^) was added to the well. After 24 h of incubation, the cells were observed by microscopy and the infected cells were recovered by centrifugation once a large number of fused cells were present. The cell pellet was lysed with 200 μL of the lysis buffer (20 mM Tris–HCl, pH7.5, 125 mM NaCl, 1 mM EDTA, 10% glycerol, 1% NP-40, 30 mM NaF, 5 mM Na_3_VO_4_) was added, followed by incubation on ice for 30 min. Then the samples were centrifuged at 15,000 rpm for 20 min at 4 °C. The supernatants (10 μL) were mixed with 3 μL of the loading buffer, incubated at 95 °C for 5 min and samples were subjected to SDS-PAGE as described below.

### Confocal microscopic analysis

Cells were cultured on poly-L-lysine-coated microscope cover glasses (Matsunami, Osaka, Japan) in a 24-well plate, fixed for 30 min with 3% formaldehyde in PBS, and permeabilized for 15 min with 0.2% Triton X-100 in PBS. The cells were then blocked with 1% of BSA in PBS for 10 min and incubated for 1 h with 5G2 mAb (200 μg/mL). The cells were washed 4 times and incubated with Alexa Fluor 488 secondary antibody for 1 h. The cells were washed 4 times and mounted onto glass slides with Prolong Gold (Invitrogen), which contains DAPI to stain the nucleus^28^.

### SDS-PAGE and western blot

The protein samples were diluted 1.3-fold with loading buffer and incubated at 95 °C for 5 min. For the CBB staining, 80–100 ng of purified sc5G2 and 25 ng each of the recombinant spike protein were loaded per lane. For the western blot, three times diluted protein samples used for the CBB staining were used. The proteins were separated on either 10% or 12% SDS Tris–glycine polyacrylamide gels. The gels were either stained with CBB or transferred to the nitrocellulose membranes. For western blot, the membranes were blocked with Blocking One reagent (Nacalai Tesque). To detect purified sc5G2 proteins, anti-MBP or anti-6xHis antibodies diluted 1:5,000 with TBS-T (0.02% Tween 20 in TBS) was added and incubated at RT for 1 h. To detect spike-bound sc5G2 proteins, 0.8 μg/mL sc5G2 proteins were added and incubated at RT for 1 h. After washing with TBS-T, anti-MBP or anti-6xHis antibodies diluted 1:5000 with TBS-T was added and incubated at RT for 1 h. A 1:5000 dilution of anti-mouse IgG-F(ab’)_2_-HRP was added and incubated at RT for 1 h.

The Vero cell lysate (10 μL of 1 × 10^6^ cell / 200 μL per lane) was loaded on the 8% gel and transferred to the nitrocellulose membranes and they were blocked with 5% skim milk (Nacalai Tesque) in TBS-T. The sc5G2 proteins (0.8 μg/mL) were added and incubated at RT for 1 h. After washing with TBS-T, anti-MBP or anti-6xHis antibodies diluted 1:5,000 with TBS-T was added, incubated at RT for 45 min and after washing anti-mouse IgG-F(ab’)_2_-HRP was added and incubated at RT for 30 min.

The 5G2 mAb (0.2 μg/mL) was used for the detection of the spike proteins in the Vero cells or the recombinant spike proteins on the membrane. The antibody was incubated with the membrane at RT for 1 h. After washing, a 1:5,000 dilution of anti-mouse IgG-F(ab’)_2_-HRP in TBS-T was added and incubated at RT for 1 h.

The membrane-bound immune complexes were detected with ECL Western Blotting Detection reagent (Cytiva) and analyzed using the ChemiDoc Touch Imaging system (BioRad).

All methods were performed in accordance with the relevant guidelines and regulations.

### Ethical statement

Animal experimental procedures were approved by the Institutional Animal Committee of Kumamoto University (ID: A2022–002) and performed by its guidelines and in compliance with the ARRIVE guidelines.

## Results

### Development of a mAb against the conserved region of the SARS-CoV-2 spike protein

**Figure 1:**
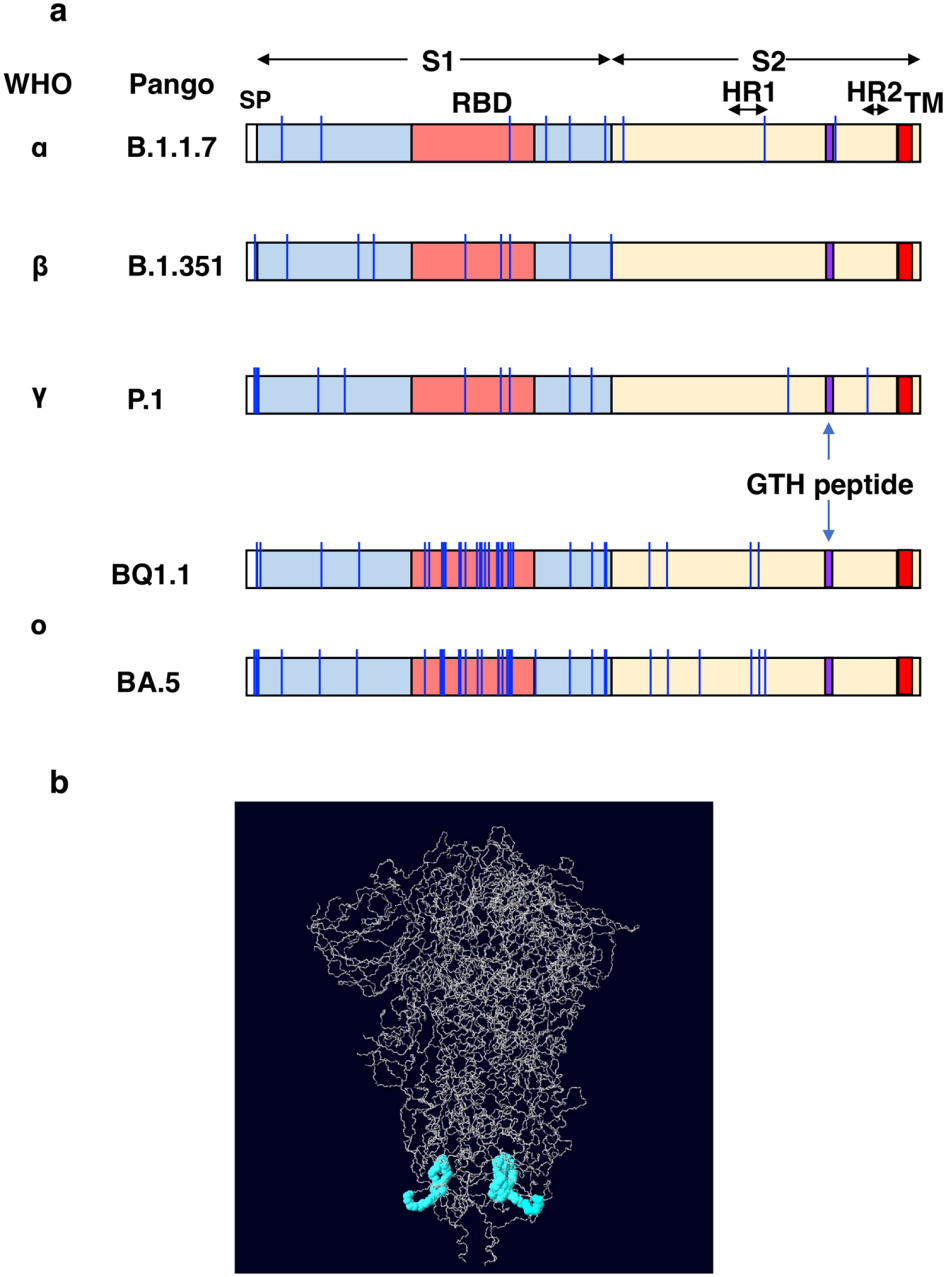
The GTH peptide on the SARS-CoV-2 spike protein. (**a**) The position of the GTH peptide in the spike proteins of the α, β, γ and o BA.5 and BQ1.1 mutant strains. The vertical blue lines show the mutation sites in the spike proteins; the purple boxes show the position of the GTH peptide. SP: signal peptide. HR1 and HR2: heptad repeat 1 and 2, respectively. TM: transmembrane domain. (**b**) The 3D structure showing the position of the GTH peptide on the spike protein. The GTH peptide shown in light blue is located in the S2 structural domain and on the surface of the spike protein. The illustration was modified from PDB 7DF3 at https://www.wwpdb.org/pdb?id=pdb_00007df3.

**Figure 2:**
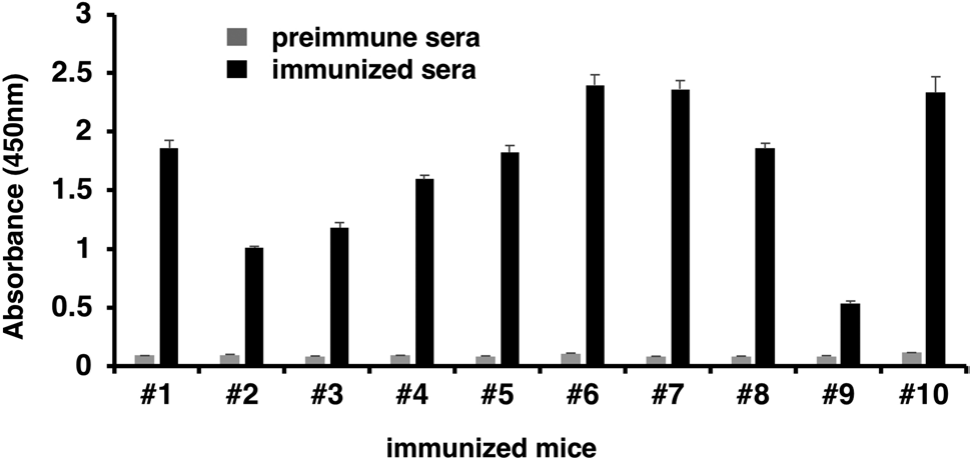
The GTH peptide-specific IgG levels in mice sera following immunization. Blood samples were collected after the mice received three subcutaneous injections of the peptide. Changes in the peptide-specific IgG levels before and after immunization of the mice were compared using ELISA. The numbers in the abscissa indicate each immunized mouse. The data represent mean ± standard error (SE).

The primary spike protein sequences of the SARS-CoV-2 strains investigated in this study were compared. Figure 1a shows the amino acid substitution sites in the spike protein of the SARS-CoV-2 variants^9^. The S1 region, particularly the receptor binding region (RBD), is associated with the most substitutions, compared with that of the S2 region. Next, we searched for epitopes located on the surface of the S2 region of the protein, which favors the binding of the antibody to infectious viral particles. We selected the GTH peptide (1099–1112: GTHWFVTQRNFYEP) that is present between the heptad repeat 1 (HR1) and HR2 regions of the S2 domain of the SARS-CoV-2 spike protein, in which no known mutations occur. Figure 1b shows that the GTH peptide is present on the surface of the spike trimer and consists of a loop structure protruding to the outside of the trimer complex. We confirmed that the peptide is predicted to bind with high affinity to MHC class II molecules, such as HLA-DR4 (HLA-DRA*01:01/HLA-DRB1*04:05) and mouse I-E^d^, but not I-A^d^ nor I-A^b^ (IEDB algorithm at http://tools.iedb.org/main/tcell/). Indeed, immunization of the GTH peptide to HLA-DR4 transgenic mice^19,29^ successfully yielded high-titer, anti-GTH peptide anti-sera (Fig. 2).

**Figure 3:**
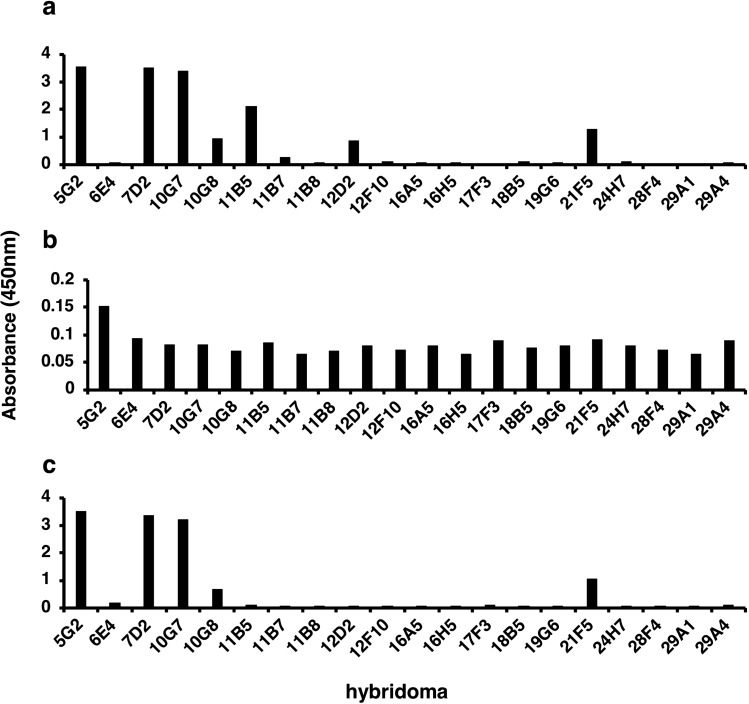
Hybridomas producing antibodies against GTH peptide, virus (WT), and recombinant S1 + S2 spike protein. The reactivities of cell culture supernatants from 20 hybridomas to (**a**) the GTH peptide, to (**b**) SARS-CoV-2 virus (WT) and to (**c**) the recombinant spike protein as determined by ELISA.

High-affinity anti-GTH peptide mAbs were obtained using GANP transgenic mice^20,30^. Cell culture supernatants from the isolated hybridomas were subjected to ELISA to test their reactivity against the antigen. One hybridoma designated 5G2 exhibited the highest affinity for the GTH peptide (Fig. 3a). We also confirmed that the cell culture supernatant from the 5G2 hybridoma reacted to the coronavirus (Fig. 3b) and recombinant spike protein (Fig. 3c). The mAb was purified using a HiTrap Protein G column and subjected to surface plasmon resonance analysis to determine the dissociation constant (*K*_d_ value) to the epitope peptide (the GTH peptide). Interestingly, the *K*_d_ value was 1.1 × 10^–10^ M, which was a relatively low *K*_d_ value compared with that of previously reported antibodies^31,32^.

**Figure 4:**
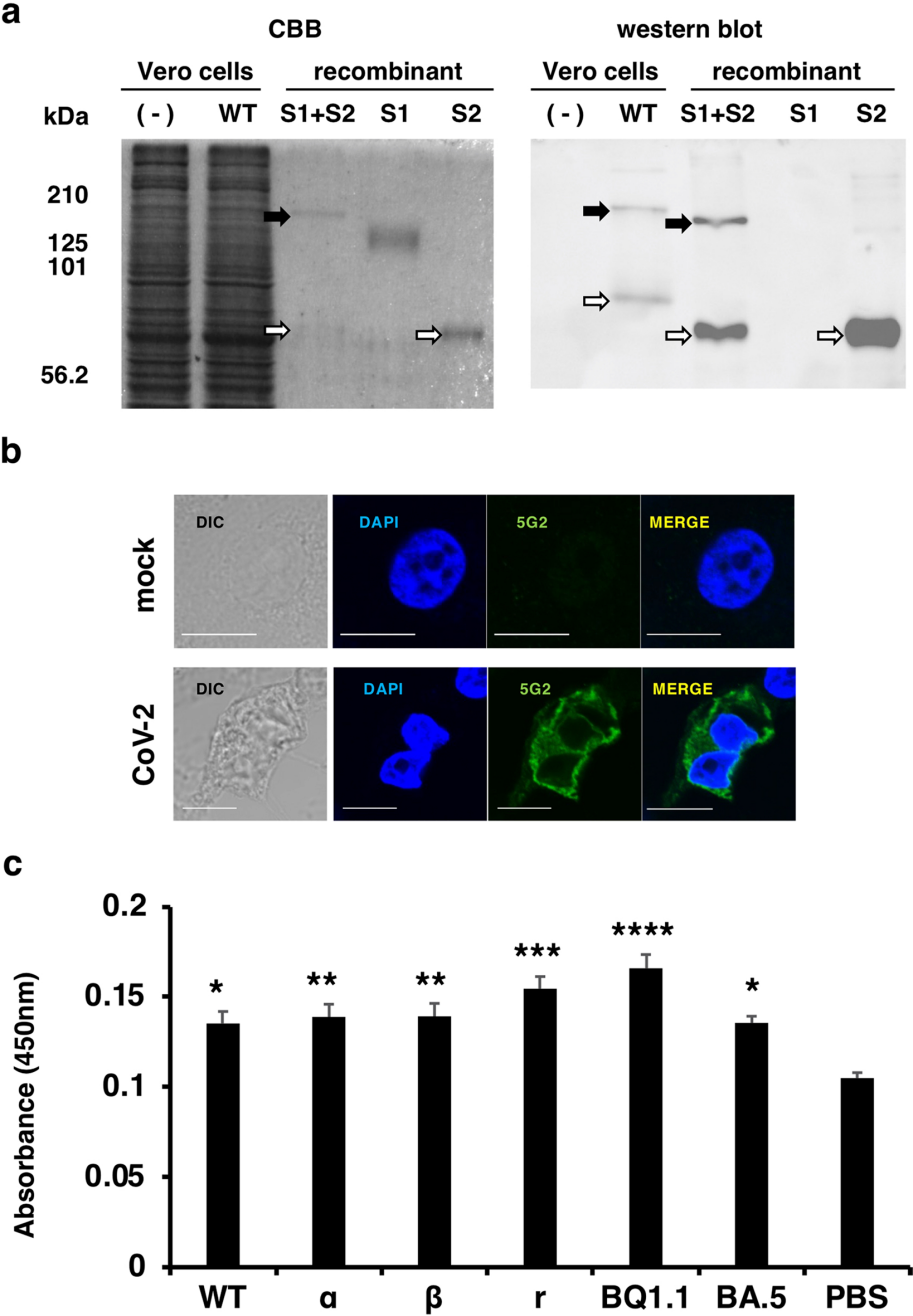
The 5G2 mAb binds to the WT and SARS-CoV-2 variants. (**a**) Binding of 5G2 mAb to SARS-CoV-2 spike proteins was detected by western blot. CBB, CBB stain (left). In the western blot (right), 0.2 μg/mL of 5G2 mAb was used and it was detected by anti-mIgG-HRP. “(-)” represents the uninfected Vero cell lysate. “WT” represents the Vero cells infected with Wuhan SARS-CoV-2. Recombinant “S1 + S2”, “S1” and “S2” stand for the recombinant spike proteins of “S1 + S2”, “S1” and “S2”, respectively. S1 + S2 and S2 proteins are indicated by the solid and open arrows, respectively. Commercially obtained recombinant S1 + S2 and S2 proteins, which lacks the cytoplasmic domain, were expressed by the Baculovirous-Insect Cell system and could be less glycosylated to show smaller molecular sizes compared to those expressed in the Vero cells. The recombinant S1 proteins produced by HEK293 cells shows larger molecular size due to the high glycosylation. (**b**) Immunofluorescence staining of spike proteins on the SARS-CoV-2 infected cell surface as detected by 5G2 mAb (green). Blue: DAPI was used for nuclear staining. Mock: Vero cells without SARS-CoV-2 infection, CoV-2: Vero cells infected with SARS-CoV-2. The leftmost panels show the phase contrast images. Scale bar: 10 μm. (**c**) ELISA for 5G2 mAb reactivity to 1 × 10^5^ of SASR-CoV-2 WT and its variants (α, β, γ, and ο of BQ1.1, and BA.5). PBS: no virus coating. The data represent mean ± standard error (SE). The statistical significance was determined by one-way analysis of variance (one-way ANOVA). *P < 0.05, **P < 0.01, ***P < 0.001, ****P < 0.0001.

Next, we determined whether the 5G2 mAb could be used for western blot, immunofluorescence microscopy, and ELISA assays. In the western blot, the 5G2 mAb specifically reacted to proteins corresponding to the molecular weight of SARS-CoV-2 spike S1 + S2 and the S2 subunit in cell lysate of the Vero cells infected with SARS-CoV-2 or the recombinant SARS-CoV-2 S1 + S2 and S2 spike proteins, but not to the lysate of the uninfected Vero cells nor the recombinant S1 protein (Fig. 4a). The protein corresponding to the molecular weight of S2 protein in the recombinant S1 + S2 sample may be the S2 subunit produced by the spontaneous cleavage of the S1 + S2 protein since it retains the S1/S2 cleavage regions. The commercial recombinant S1 + S2 protein and S2 protein lack the cytoplasmic regions and were produced by the Baculovirous-Insect Cell system, that may have shorter sugar chains compared to those expressed in the Vero cells. Therefore, the S1 + S2 and the S2 proteins expressed in the Vero cells and the recombinant proteins may show different molecular sizes.

The 5G2 mAb stained the cell surface and cytosol of SARS-CoV-2-infected Vero cells, but not uninfected cells (Fig. 4b). Moreover, an ELISA revealed that the mAb reacted with the original Wuhan strain (WT) as well as its variants, including α, β, γ, and ο of BQ1.1, and BA.5 with significantly higher A_450_ values (Fig. 4c). These data indicate that the mAb is useful for western blot, immunofluorescence, and ELISA.

### Variable regions of the 5G2 mAb

**Figure 5:**
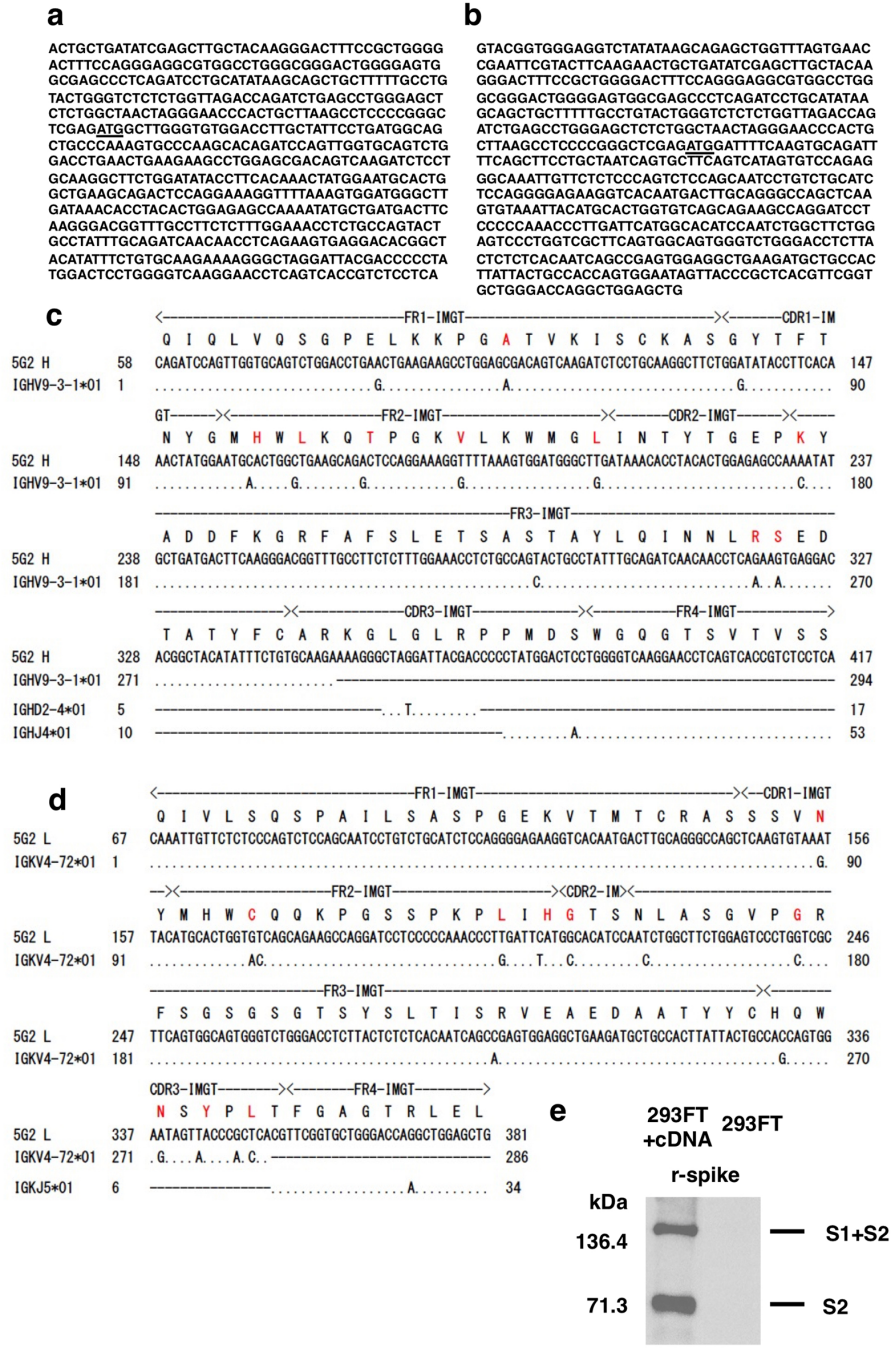
cDNA sequence of the variable regions of the 5G2 mAb. (**a**,**b**) cDNA sequences of the variable regions of the heavy (**a**) and the light (**b**) chains of 5G2. The underlined ATG sequence indicates the predicted start codons. (**c**,**d**) Predicted use of the V, (D), and J segments of the 5G2 heavy (**c**) and the light (**d**) chain sequences. The nucleic acid substitutions possibly resulting from the somatic hypermutation and the amino acid substitutions (red) are indicated. (**e**) Culture supernatant (1 mL) of HEK293FT cells in five 10 cm culture dishes transfected with the 5G2 heavy and light chain cDNAs (293FT + cDNA), but not that of HEK293FT cells without transfection (293FT), showed the bands corresponding to the S1 + S2 and S2 subunits of the recombinant spike protein (r-spike) by western blot. The bound recombinant 5G2 was detected by the anti-mIgG-HRP as described in the "Materials and methods" section.

Total RNA was extracted from the 5G2 hybridoma and the cDNAs encoding the variable regions of the heavy and light chains were cloned using a simplified 5'-RACE^22^ and RT-PCR (Fig. 5a,b). The cDNA sequence of the heavy chain suggests that the clone consisted of IGHV9-3–1*01, IGHD2-4*01, and IGHJ4*01 with 12 mutations, which resulted in nine amino acid substitutions in the VDJ region (Fig. 5c). Similarly, the variable region of the light chain was predicted to consist of IGKV4-72*01 and IGKJ5*01 with 15 mutations, resulting in 11 amino acid substitutions in the VJ region (Fig. 5d).

The cloned heavy and light chain cDNAs were fused to the cDNAs of the mouse IgG constant γ1 and κ chains, respectively (Supplementary Fig. 1a and b) and the cDNAs were transfected into HEK293FT cells. The culture supernatant from cDNA-transfected HEK293FT cells specifically detected the S1 + S2 and S2 subunits of recombinant SARS-CoV-2 spike protein, but not the S1 subunit, as the 5G2 mAb did (Fig. 5e). These data suggest that the cloned variable regions bind to the epitope.

### Single-chain form of the 5G2

Previous studies have indicated that the single-chain variable antibody fragment (scFv) exhibits a small molecular weight (about 1/6 of the whole antibody molecule)^33^ and can even be expressed in yeast and plant cells^34,35^. scFv spontaneously folds and forms a natural structure. Thus, it possesses antigen-binding properties with the prospect of broad applications^36^, such as therapy for antiviral, tumor, autoimmune diseases, and targeted drugs^37,38^.

**Figure 6:**
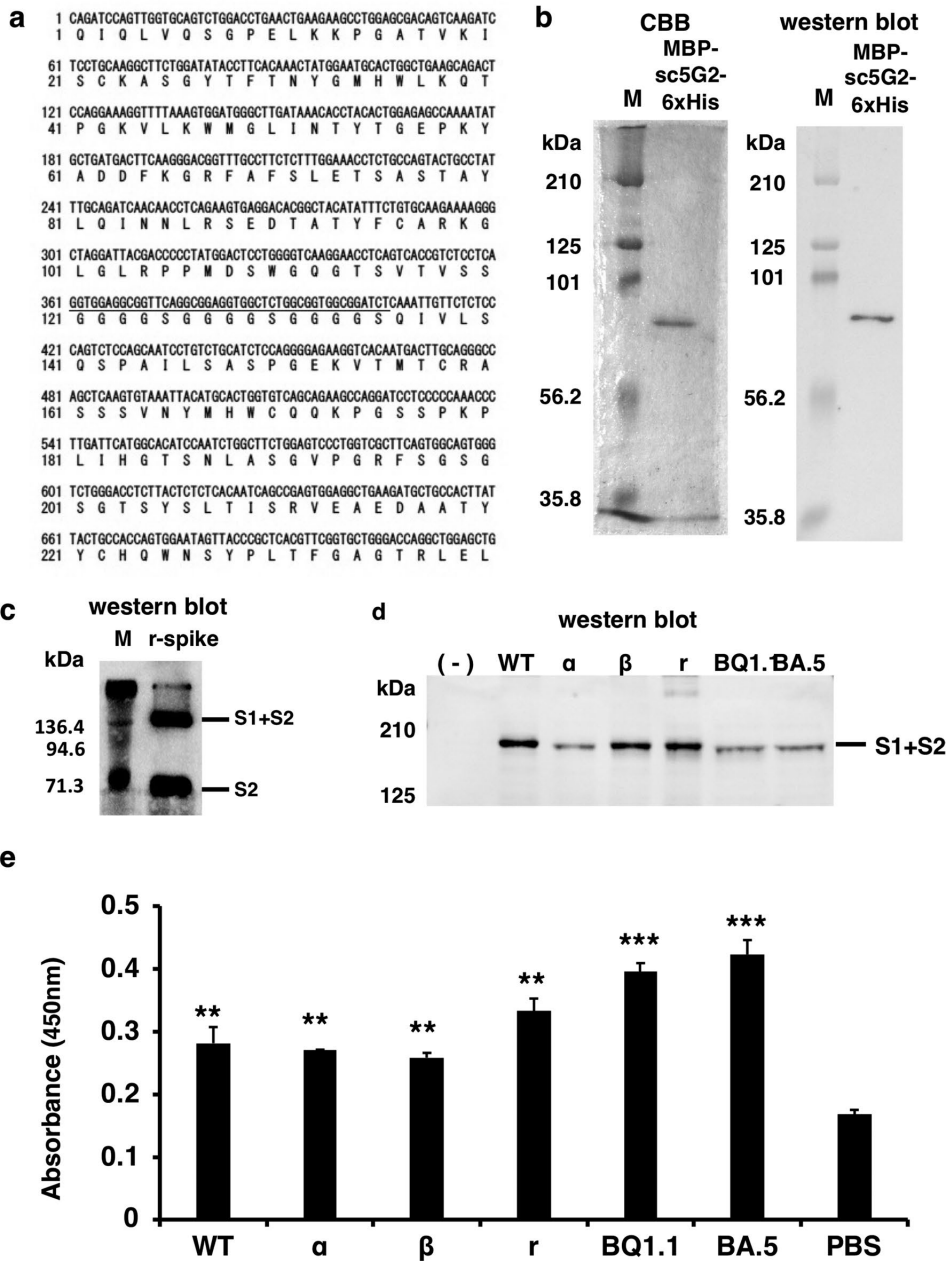
MBP-sc5G2-6xHis produced from HEK293FT cells. (**a**) cDNA sequence of MBP-sc5G2-6xHis. The underlined DNA sequence encodes the (G_4_S)_3_ linker sequence. (**b**) MBP-sc5G2-6xHis produced by HEK293FT was subjected to SDS-PAGE. The proteins were stained with CBB (left) and analyzed by western blot using anti-MBP antibody and anti-mIgG-HRP (right). M: Molecular weight marker. (**c**) MBP-sc5G2-6xHis produced by HEK293FT (0.8 μg/mL) was confirmed to bind specifically to the S1 + S2 and the S2 subunit of the recombinant spike protein by western blot. The bound MBP-sc5G2-6xHis was detected by the anti-MBP antibody and anti-mIgG-HRP. M: Molecular weight marker. (**d**) MBP-sc5G2-6xHis produced by HEK293FT detects the S1 + S2 spike proteins in the Vero cells infected with SARS-CoV-2 WT and its variants, α, β, γ, BQ1.1, and BA.5 but not in the uninfected Vero cells (-) as confirmed by western blot using MBP-sc5G2-6xHis (0.8 μg/mL), anti-MBP antibody and anti-mIgG-HRP. (**e**) ELISA for MBP-sc5G2-6xHis reactivity to 1 × 10^5^ of SASR-CoV-2 WT and its variants (α, β, γ, and ο of BQ1.1, and BA.5). PBS: no virus coating. The data represent mean ± standard error (SE). The statistical significance was determined by one-way analysis of variance (one-way ANOVA). **P < 0.01, ***P < 0.001.

To generate the single-chain form of 5G2 (sc5G2), the cDNAs encoding the heavy and light chains were fused with a DNA linker encoding three G_4_S repeats (Fig. 6a). The sc5G2 cDNA was then fused to a cDNA fragment encoding the MBP at the 5' end of the sc5G2 cDNA to stabilize its expression and facilitate purification. A 6xHis-tag sequence was also inserted at the 3' end of the sc5G2 cDNA to further facilitate the purification of the recombinant protein. Thus, we obtained a cDNA clone encoding the MBP-sc5G2-6xHis protein.

The MBP-sc5G2-6xHis cDNA was transfected into HEK293FT cells and the protein was purified using HisTrap and MBPTrap columns from cell culture supernatants (Fig. 6b). The purified MBP-sc5G2-6xHis detected the S1 + S2 and S2 subunits of the recombinant SARS-CoV-2 spike protein (Fig. 6c), similar to the 5G2 mAb (Fig. 4a) and the recombinant heavy and light chains of 5G2 (Fig. 5e).

The MBP-sc5G2-6xHis from HEK293FT cells detected S1 + S2 subunits of SARS-CoV-2 WT and the variant strains of α, β, r, and o of BQ1.1 and BA.5 as determined by the western blot (Fig. 6d) and the viral particles by ELISA with significantly higher A_450_ values (Fig. 6e). These data indicate that sc5G2 binds to the same epitope of the mAb 5G2.

We also tried to obtain MBP-free sc5G2 from HEK293FT cells, however, only a very small amount of protein was obtained. It was considered that the addition of the MBP tag greatly increased the amount of MBP-sc5G2-6xHis in HEK293FT cells.

### Preparation of sc5G2 in *E. coli*

**Figure 7:**
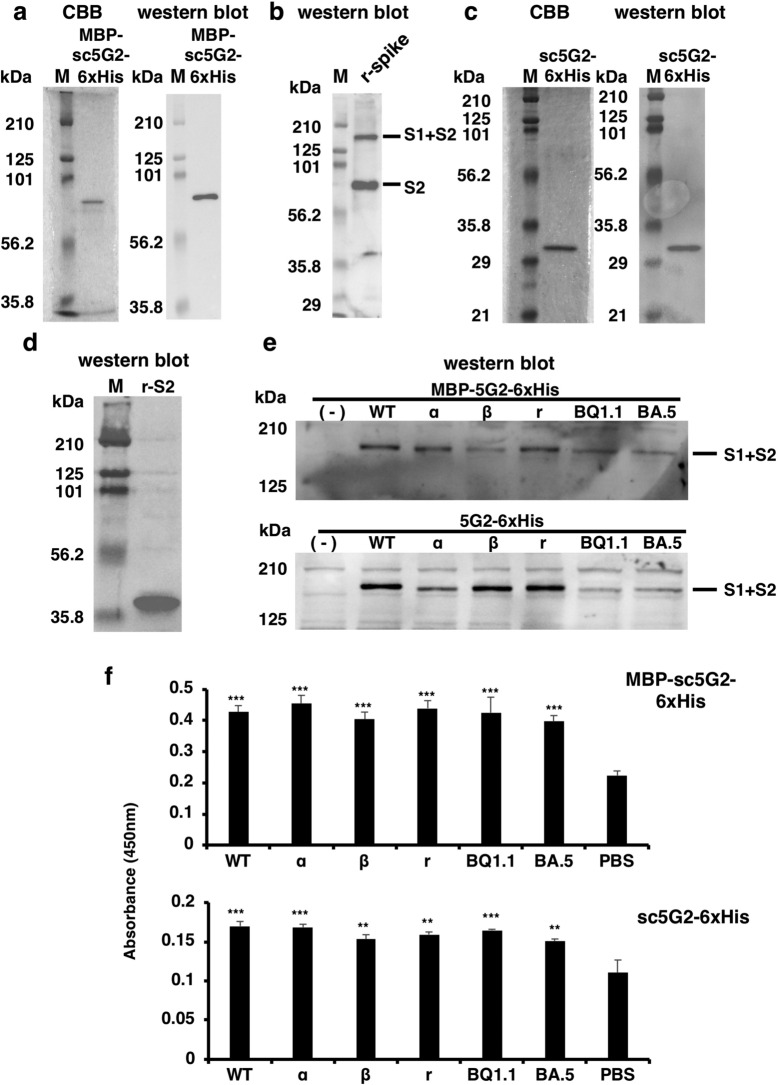
Production of sc5G2s by *E. coli*. (**a**) Refolded MBP-sc5G2-6xHis produced by *E. coli* was subjected to SDS-PAGE. The proteins were stained with CBB (left) and analyzed by western blot using anti-MBP antibody and anti-mIgG-HRP (right). M: Molecular weight marker. (**b**) MBP-sc5G2-6xHis (0.8 μg/mL) produced by *E. coli* was used to detect recombinant S1 + S2 and the S2 proteins by western blot as in (a). M: Molecular weight marker. (**c**) Refolded sc5G2-6xHis protein produced by *E. coli* was subjected to SDS-PAGE. The proteins were stained with CBB (left) and analyzed by western blot using anti-6xHis antibody and anti-mIgG-HRP (right). M: Molecular weight marker. (**d**) sc5G2-6xHis (0.8 μg/mL) produced by *E. coli* was used to detect the recombinant S2 subunit of the spike protein without 6xHis (r-S2) by western blot using anti-6xHis antibody and anti-mIgG-HRP. M: Molecular weight marker. (**e**) MBP-sc5G2-6xHis (0.8 μg/mL, top) and sc5G2-6xHis (0.8 μg/mL, bottom) produced by *E. coli* were used to detect the S1 + S2 of the spike proteins in the Vero cells infected with SARS-CoV-2 WT as well as the variants, α, β, γ, BQ1.1, and BA.5 by western blot using anti-MBP antibody (top) or anti-6xHis antibody (bottom) and anti-mIgG-HRP. (-): Vero cells only. M: Molecular weight marker. (**f**) MBP-sc5G2-6xHis (0.8 μg/mL, top) and sc5G2-6xHis (0.8 μg/mL, bottom) produced by *E. coli* was used to detect particles of SARS-CoV-2 WT as well as the variants, α, β, γ, BQ1.1, and BA.5 by ELISA. PBS: no virus coated. The data represent mean ± standard error (SE). The statistical significance was determined by one-way analysis of variance (one-way ANOVA). **P < 0.01, ***P < 0.001.

Because scFvs can be expressed as a functional protein even in *E. coli* cells^39,40^, we determined whether the MBP-sc5G2-6xHis protein could be obtained using *E. coli* cells. MBP-sc5G2-6xHis was expressed in the BL21(DE3) strain; however, most of the protein was present in the insoluble fraction, thus we attempted to refold the protein by gradually reducing the concentration of GdnHCl. The insoluble pellet was suspended in the 6 M GdnHCl and 20 mM imidazole in TBS and applied to a HisTrap column and the bound proteins were recovered as described in the “Materials and methods” section. After stepwise dialysis, the soluble protein fraction was applied to an MBPTrap column and the purified MBP-sc5G2-6xHis protein was obtained (Fig. 7a).

The MBP-sc5G2-6xHis protein isolated from *E. coli* detected the S1 + S2 and S2 subunits of the recombinant SARS-CoV-2 spike proteins (Fig. 7b) and S1 + S2 expressed in Vero cells infected with SARS-CoV-2 WT, and the variant strains, α, β, r, and ο of BQ1.1 and BA.5 (Fig. 7e top). These data indicate that the functional MBP-sc5G2-6xHis can be obtained from *E. coli*. MBP-sc5G2-6xHis prepared from *E. coli* also reacted viral particles as revealed by ELISA with significantly higher A_450_ values (Fig. 7f top). These data indicate that MBP-sc5G2-6xHis has the same function to that of the 5G2 mAb.

Finally, we determined whether the MBP tag is required to obtain functional sc5G2 protein. We constructed the sc5G2-6xHis cDNA, which does not encode MBP, and *E. coli* BL21(DE3) cells were transformed with the cDNA-encoding vector. Although the sc5G2-6xHis protein was much less produced by *E. coli* compared to the MBP-sc5G2-6xHis (Supplementary Fig. 2) and was included in the insoluble fraction, we succeeded in refolding the sc5G2-6xHis protein by the similar procedure for the MBP-sc5G2-6xHis refolding. Purified sc5G2-6xHis exhibited the expected molecular weight of approximately 30 kDa (Fig. 7c) and reacted with the recombinant S2 subunit without 6xHis tag (Fig. 7d). sc5G2-6xHis detected the S1 + S2 subunits in cell lysates from Vero cells infected with SARS-CoV-2 WT and variant strains, α, β, r, and ο of BQ1.1 and BA.5 (Fig. 7e bottom). sc5G2-6xHis also detected viral particles of SARS-CoV-2 WT and the variant strains in the ELISA with significantly higher A_450_ values (Fig. 7f bottom). These data indicate that the sc5G2-6xHis without MBP domain folded properly after denaturation and showed expected specificity to the WT and variant spike proteins on the membrane as well as those on the viral particles.

## Discussion

We generated a mAb specific to the S2 subunit of the SARS-CoV-2 spike protein and produced single-chain forms of 5G2. Similar to the original 5G2 mAb, the recombinant sc5G2 specifically detected the S1 + S2 and S2 subunits of the SARS-CoV-2 spike protein as well as the GTH peptide of the S2 subunit. The sc5G2 reacted not only to the original SARS-CoV-2 strain, but also to several variants (α, β, r and o). This is consistent with the fact that the amino acid sequences of the GTH peptide (GTHWFVTQRNFYEP) are conserved among the spike proteins of the original virus and the variants investigated in this study. Thus, it is expected that the sc5G2 will be useful for detecting any SARS-CoV-2 variants if they do not have the mutations in the GTH peptide region.

Although the 5G2 mAb and its single-chain variants can detect both the GTH peptide and viral particles, the reactivity towards viral particles was lower compared to that of the GTH peptide in the ELISA. It is possible that the weakened response was caused by a difference in copy number of the epitope between the peptide and viral particle samples. In the ELISA, we coated the wells with 50 μL of the GTH peptide (40 ng/mL)/well. The molecular weight of the GTH peptide is 1782 Da; thus, the number of GTH peptides in the 50 μL of coating solution was calculated as approximately 6.8 × 10^11^. On the other hand, we coated the wells with viral particles at 1 × 10^5^ /well for the ELISA. There are approximately 24 ± 9 spike trimers per virion^41^; therefore, only 1 × 10^7^ epitopes at most were likely coated on the bottom of the wells, which is considerably fewer compared with the number of GTH peptide molecules.

In western blot, the spike proteins of the Omicron variants BQ1.1 and BA.5 were weakly detected by sc5G2 compared to those of WT and α, β, r variants. It is reported that Omicron variants replicate to lower titers compared with WT^42^ and with α and r variants^43^ after 24 h of infection to the Vero cells. Recently, it is reported that the Omicron variants were optimized for replication in the upper respiratory tract^44^ and thus they might replicate slower in Vero cells compared to other variants do. These observations could explain the weak reactivity of sc5G2 to the spike proteins in the Vero cells infected with the Omicron variants compared to those infected with Wuhan strain and α, β, r variants. On the other hand, the same numbers of each viral strain were coated on the ELISA plate and sc5G2s could detect Omicron variants at comparable OD levels to those of Wuhan strain and α, β, r variants in ELISA.

MBP-sc5G2-6xHis was successfully expressed in human HEK293FT cells. Although this protein was included in the insoluble fraction of *E. coli*, the protein could be refolded appropriately following the denaturing steps. At first, we introduced the MBP tag for stable expression, improved solubility and to facilitate the purification of the sc5G2, however, we found that the sc5G2 could be refolded and reacted to antigens even without the MBP tag. This indicates that while addition of MBP tag much improved the amount of expressed MBP-sc5G2-6xHis, sc5G2 fragment itself is to some extent stable and can be correctly refolded in vitro .

Because the epitope of the sc5G2 is conserved among variants, sc5G2 is expected to be useful for the development of a universal test kit for COVID-19 that instantly detects any variant strain of SARS-CoV-2 like a flu test kit.

The 5G2 mAb, MBP-sc5G2-6xHis and sc5G2-6xHis did not show the neutralization activity against the SARS-CoV-2 infection to the Vero cells, most likely due to that the epitope locates far away from the RBD of the spike protein. However, sc5G2-6xHis has a small MW of 28.2 kDa that retains the reactivity to the spike proteins of the all SARS-CoV-2 variants examined and could be prepared by the cost-effective bacterial cell expression system. Therefore, the sc5G2 will be a useful platform for the development of the antibody–drug conjugate such as gemtuzumab ozogamicin^45^ and for the bispecific antibodies that link infected cells and cytotoxic immune cells^46,47^. The spike protein heptad repeats 1 and 2 (HR1 and HR2) play an important role for the host and viral membrane fusion and the 5-Helix peptide consisting of three HR1 and two HR2 fragments was reported to interfere the membrane fusion^48^. Considering that the 5G2 epitope is located between HR1 and HR2, the sc5G2 would deliver the 5-Helix peptide to the point of membrane fusion more effectively. We hope that the genetic and amino acid sequence information of 5G2 and sc5G2 will be helpful for researchers to develop anti-Covid-19 therapy reagents.

### Supplementary Information

Supplementary Figures.

## Data Availability

All data generated (including supplementary data) and analyzed during this study are included in this paper. The DNA sequence data are accessible in DDBJ (https://www.ddbj.nig.ac.jp/index-e.html) database.
