# Supplementary material for: Development of a single-chain variable antibody fragment against a conserved region of the SARS-CoV-2 spike protein
Source: Sci Rep. 2024 Jun 22;14:14419. doi: 10.1038/s41598-024-64103-7 (PMC11193732; doi:10.1038/s41598-024-64103-7)
Supplement: Supplementary file 1 — Supplementary Figures. [file 41598_2024_64103_MOESM1_ESM.pptx]

## Slide 1
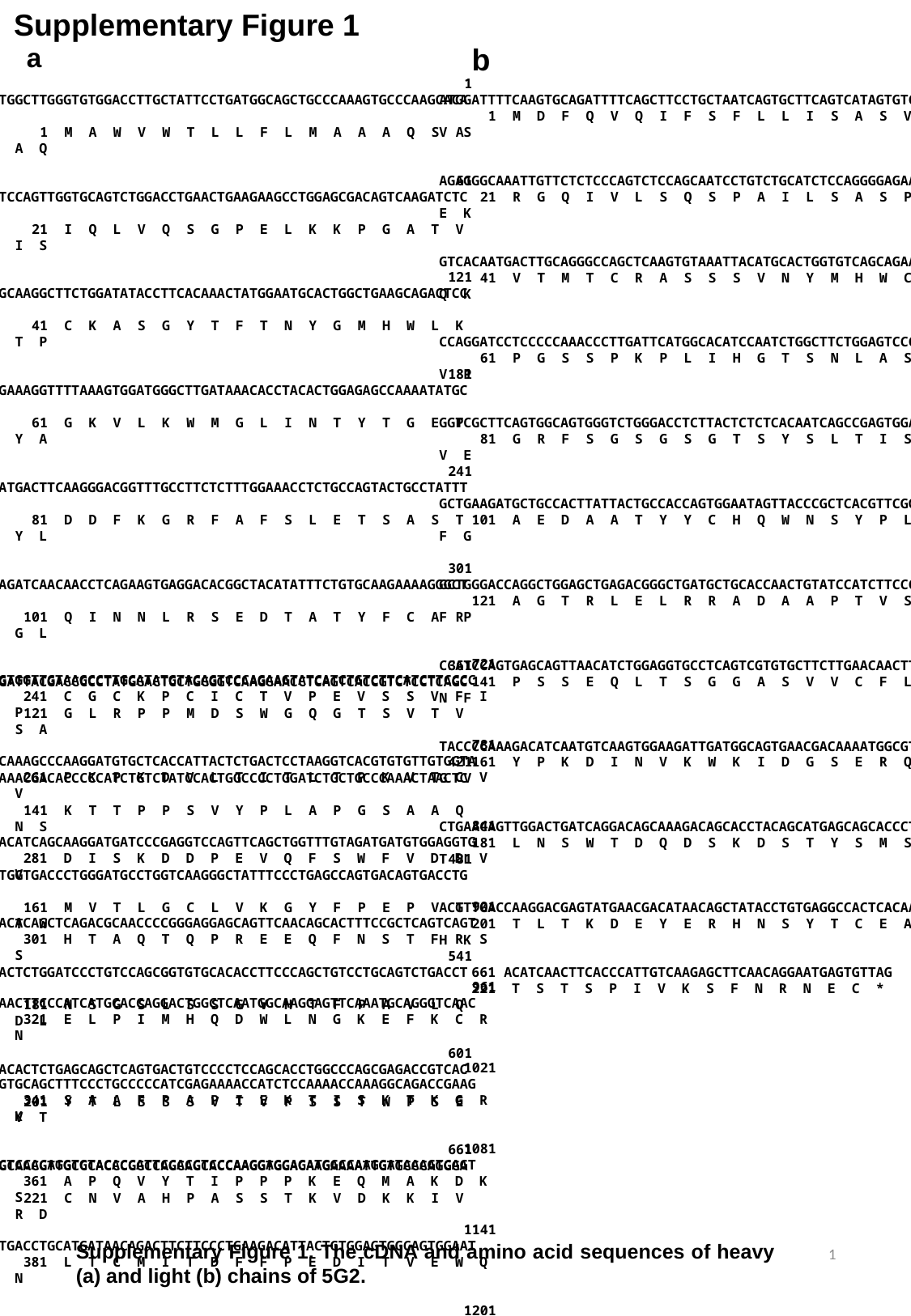

Supplementary Figure 1
a
b
 1 ATGGCTTGGGTGTGGACCTTGCTATTCCTGATGGCAGCTGCCCAAAGTGCCCAAGCACAG
 1 M A W V W T L L F L M A A A Q S A Q A Q
 61 ATCCAGTTGGTGCAGTCTGGACCTGAACTGAAGAAGCCTGGAGCGACAGTCAAGATCTCC
 21 I Q L V Q S G P E L K K P G A T V K I S
 121 TGCAAGGCTTCTGGATATACCTTCACAAACTATGGAATGCACTGGCTGAAGCAGACTCCA
 41 C K A S G Y T F T N Y G M H W L K Q T P
 181 GGAAAGGTTTTAAAGTGGATGGGCTTGATAAACACCTACACTGGAGAGCCAAAATATGCT
 61 G K V L K W M G L I N T Y T G E P K Y A
 241 GATGACTTCAAGGGACGGTTTGCCTTCTCTTTGGAAACCTCTGCCAGTACTGCCTATTTG
 81 D D F K G R F A F S L E T S A S T A Y L
 301 CAGATCAACAACCTCAGAAGTGAGGACACGGCTACATATTTCTGTGCAAGAAAAGGGCTA
 101 Q I N N L R S E D T A T Y F C A R K G L
 361 GGATTACGACCCCCTATGGACTCCTGGGGTCAAGGAACCTCAGTCACCGTCTCCTCAGCC
 121 G L R P P M D S W G Q G T S V T V S S A
 421 AAAACGACACCCCCATCTGTCTATCCACTGGCCCCTGGATCTGCTGCCCAAACTAACTCC
 141 K T T P P S V Y P L A P G S A A Q T N S
 481 ATGGTGACCCTGGGATGCCTGGTCAAGGGCTATTTCCCTGAGCCAGTGACAGTGACCTGG
 161 M V T L G C L V K G Y F P E P V T V T W
 541 AACTCTGGATCCCTGTCCAGCGGTGTGCACACCTTCCCAGCTGTCCTGCAGTCTGACCTC
 181 N S G S L S S G V H T F P A V L Q S D L
 601 TACACTCTGAGCAGCTCAGTGACTGTCCCCTCCAGCACCTGGCCCAGCGAGACCGTCACC
 201 Y T L S S S V T V P S S T W P S E T V T
 661 TGCAACGTTGCCCACCCGGCCAGCAGCACCAAGGTGGACAAGAAAATTGTGCCCAGGGAT
 221 C N V A H P A S S T K V D K K I V P R D
 1 ATGGATTTTCAAGTGCAGATTTTCAGCTTCCTGCTAATCAGTGCTTCAGTCATAGTGTCC
 1 M D F Q V Q I F S F L L I S A S V I V S
 61 AGAGGGCAAATTGTTCTCTCCCAGTCTCCAGCAATCCTGTCTGCATCTCCAGGGGAGAAG
 21 R G Q I V L S Q S P A I L S A S P G E K
 121 GTCACAATGACTTGCAGGGCCAGCTCAAGTGTAAATTACATGCACTGGTGTCAGCAGAAG
 41 V T M T C R A S S S V N Y M H W C Q Q K
 181 CCAGGATCCTCCCCCAAACCCTTGATTCATGGCACATCCAATCTGGCTTCTGGAGTCCCT
 61 P G S S P K P L I H G T S N L A S G V P
 241 GGTCGCTTCAGTGGCAGTGGGTCTGGGACCTCTTACTCTCTCACAATCAGCCGAGTGGAG
 81 G R F S G S G S G T S Y S L T I S R V E
 301 GCTGAAGATGCTGCCACTTATTACTGCCACCAGTGGAATAGTTACCCGCTCACGTTCGGT
 101 A E D A A T Y Y C H Q W N S Y P L T F G
 361 GCTGGGACCAGGCTGGAGCTGAGACGGGCTGATGCTGCACCAACTGTATCCATCTTCCCA
 121 A G T R L E L R R A D A A P T V S I F P
 421 CCATCCAGTGAGCAGTTAACATCTGGAGGTGCCTCAGTCGTGTGCTTCTTGAACAACTTC
 141 P S S E Q L T S G G A S V V C F L N N F
 481 TACCCCAAAGACATCAATGTCAAGTGGAAGATTGATGGCAGTGAACGACAAAATGGCGTC
 161 Y P K D I N V K W K I D G S E R Q N G V
 541 CTGAACAGTTGGACTGATCAGGACAGCAAAGACAGCACCTACAGCATGAGCAGCACCCTC
 181 L N S W T D Q D S K D S T Y S M S S T L
 601 ACGTTGACCAAGGACGAGTATGAACGACATAACAGCTATACCTGTGAGGCCACTCACAAG
 201 T L T K D E Y E R H N S Y T C E A T H K
 661 ACATCAACTTCACCCATTGTCAAGAGCTTCAACAGGAATGAGTGTTAG
 221 T S T S P I V K S F N R N E C *
 721 TGTGGTTGTAAGCCTTGCATATGTACAGTCCCAGAAGTATCATCTGTCTTCATCTTCCCC
 241 C G C K P C I C T V P E V S S V F I F P
 781 CCAAAGCCCAAGGATGTGCTCACCATTACTCTGACTCCTAAGGTCACGTGTGTTGTGGTA
 261 P K P K D V L T I T L T P K V T C V V V
 841 GACATCAGCAAGGATGATCCCGAGGTCCAGTTCAGCTGGTTTGTAGATGATGTGGAGGTG
 281 D I S K D D P E V Q F S W F V D D V E V
 901 CACACAGCTCAGACGCAACCCCGGGAGGAGCAGTTCAACAGCACTTTCCGCTCAGTCAGT
 301 H T A Q T Q P R E E Q F N S T F R S V S
 961 GAACTTCCCATCATGCACCAGGACTGGCTCAATGGCAAGGAGTTCAAATGCAGGGTCAAC
 321 E L P I M H Q D W L N G K E F K C R V N
 1021 AGTGCAGCTTTCCCTGCCCCCATCGAGAAAACCATCTCCAAAACCAAAGGCAGACCGAAG
 341 S A A F P A P I E K T I S K T K G R P K
 1081 GCTCCACAGGTGTACACCATTCCACCTCCCAAGGAGCAGATGGCCAAGGATAAAGTCAGT
 361 A P Q V Y T I P P P K E Q M A K D K V S
 1141 CTGACCTGCATGATAACAGACTTCTTCCCTGAAGACATTACTGTGGAGTGGCAGTGGAAT
 381 L T C M I T D F F P E D I T V E W Q W N
 1201 GGGCAGCCAGCGGAGAACTACAAGAACACTCAGCCCATCATGGACACAGATGGCTCTTAC
 401 G Q P A E N Y K N T Q P I M D T D G S Y
 1261 TTCGTCTACAGCAAGCTCAATGTGCAGAAGAGCAACTGGGAGGCAGGAAATACTTTCACC
 421 F V Y S K L N V Q K S N W E A G N T F T
 1321 TGCTCTGTGTTACATGAGGGCCTGCACAACCACCATACTGAGAAGAGCCTCTCCCACTCT
 441 C S V L H E G L H N H H T E K S L S H S
 1381 CCTGGTAAATGA
 461 P G K *
1
Supplementary Figure 1. The cDNA and amino acid sequences of heavy (a) and light (b) chains of 5G2.

## Slide 2
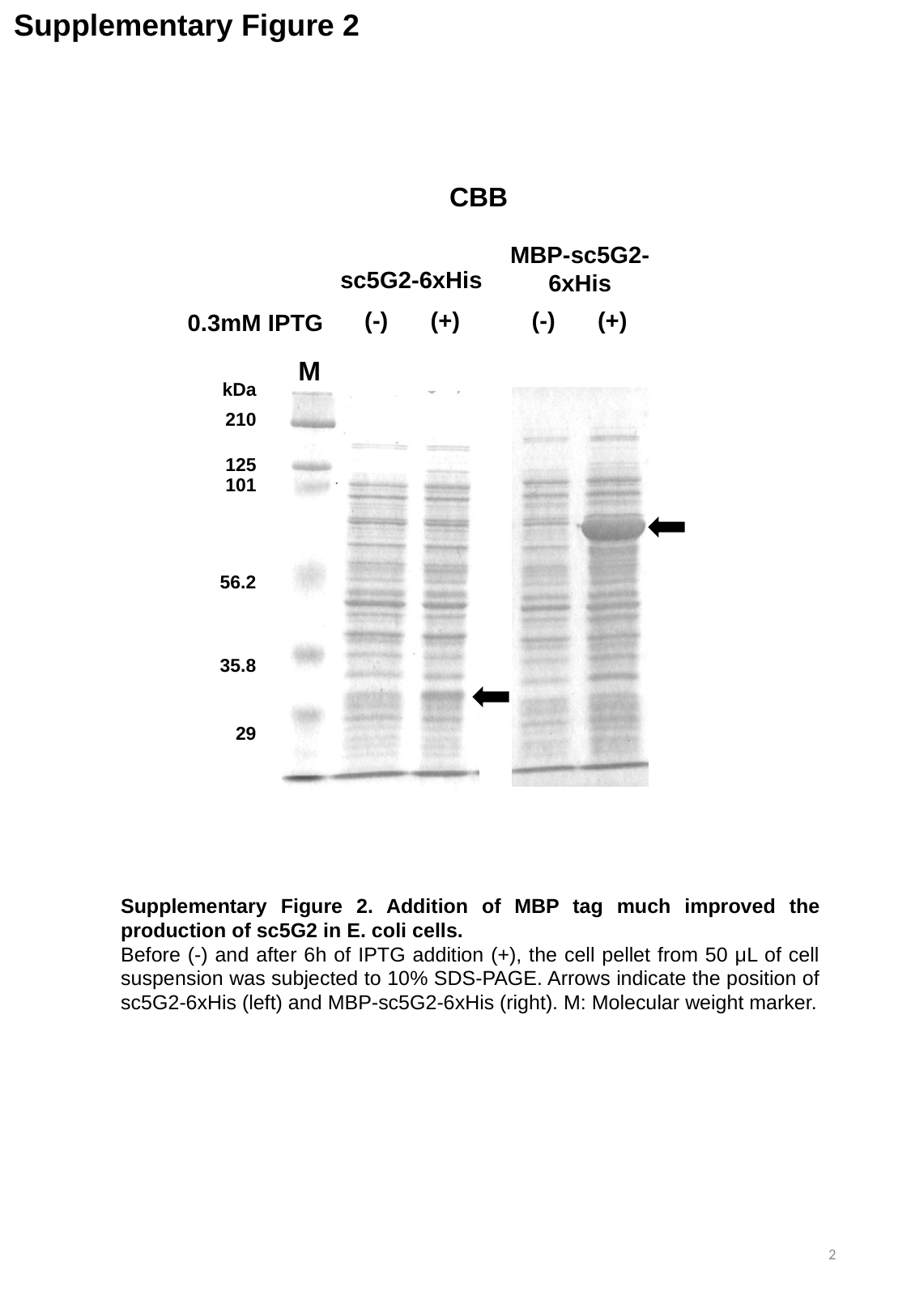

Supplementary Figure 2
CBB
MBP-sc5G2-6xHis
sc5G2-6xHis
(-)
(+)
(-)
(+)
0.3mM IPTG
M
kDa
210
125
101
56.2
35.8
29
Supplementary Figure 2. Addition of MBP tag much improved the production of sc5G2 in E. coli cells.
Before (-) and after 6h of IPTG addition (+), the cell pellet from 50 μL of cell suspension was subjected to 10% SDS-PAGE. Arrows indicate the position of sc5G2-6xHis (left) and MBP-sc5G2-6xHis (right). M: Molecular weight marker.
2

## Slide 3
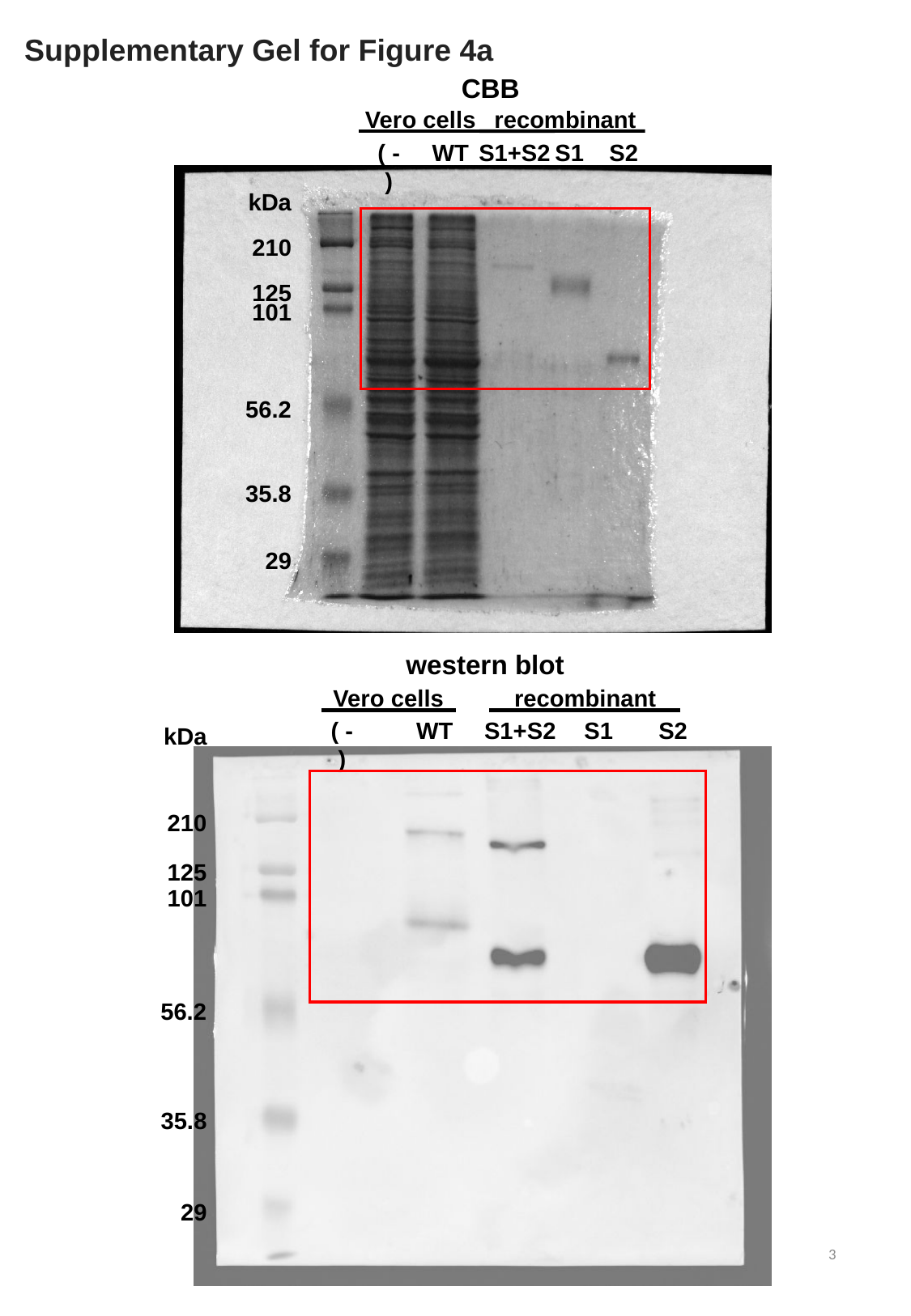

Supplementary Gel for Figure 4a
CBB
Vero cells
recombinant
( - )
WT
S1+S2
S1
S2
kDa
210
125
101
56.2
35.8
29
western blot
Vero cells
recombinant
( - )
WT
S1+S2
S1
S2
kDa
210
125
101
56.2
35.8
29
3

## Slide 4
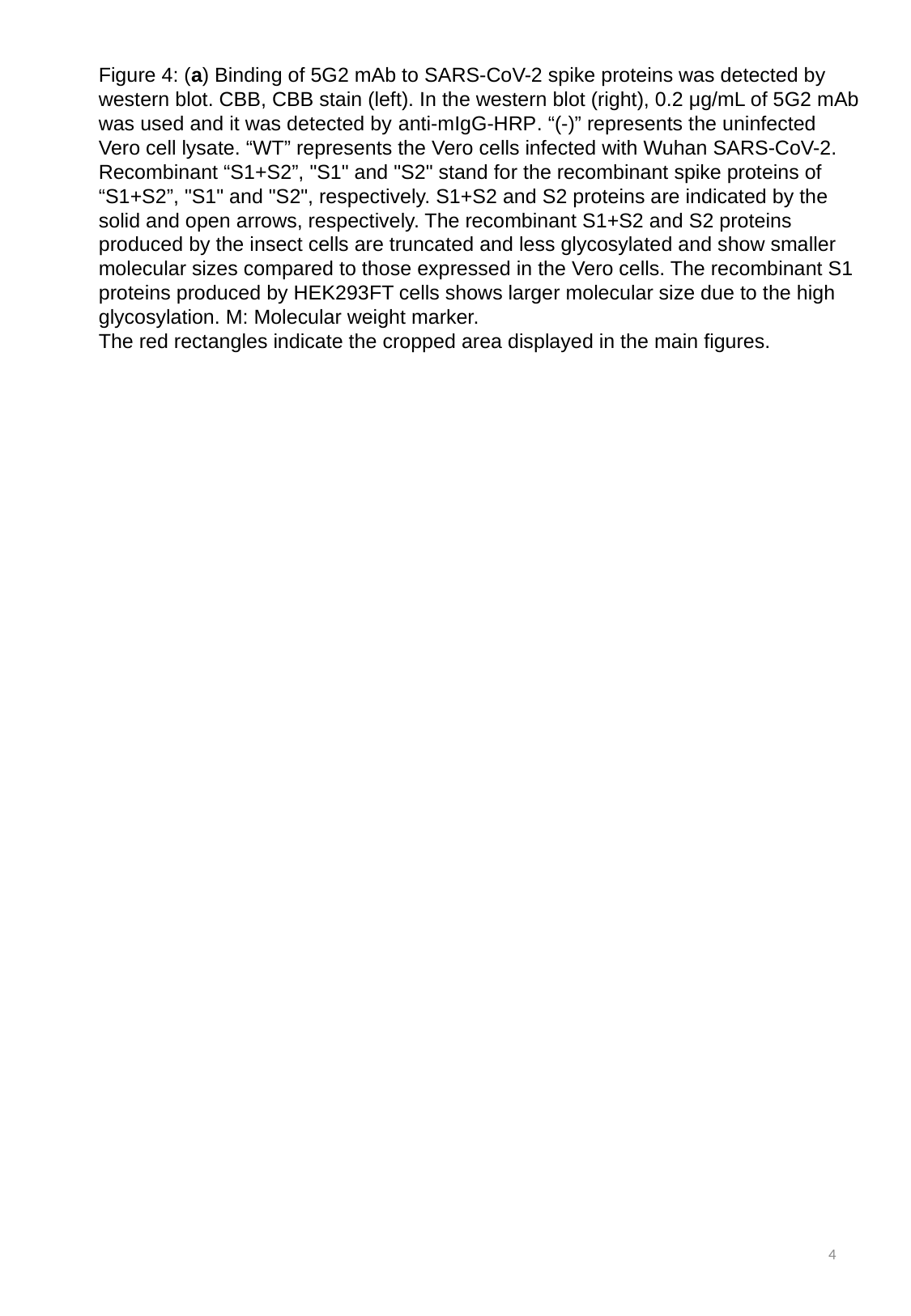

Figure 4: (a) Binding of 5G2 mAb to SARS-CoV-2 spike proteins was detected by western blot. CBB, CBB stain (left). In the western blot (right), 0.2 μg/mL of 5G2 mAb was used and it was detected by anti-mIgG-HRP. “(-)” represents the uninfected Vero cell lysate. “WT” represents the Vero cells infected with Wuhan SARS-CoV-2. Recombinant “S1+S2”, "S1" and "S2" stand for the recombinant spike proteins of “S1+S2”, "S1" and "S2", respectively. S1+S2 and S2 proteins are indicated by the solid and open arrows, respectively. The recombinant S1+S2 and S2 proteins produced by the insect cells are truncated and less glycosylated and show smaller molecular sizes compared to those expressed in the Vero cells. The recombinant S1 proteins produced by HEK293FT cells shows larger molecular size due to the high glycosylation. M: Molecular weight marker.
The red rectangles indicate the cropped area displayed in the main figures.
4

## Slide 5
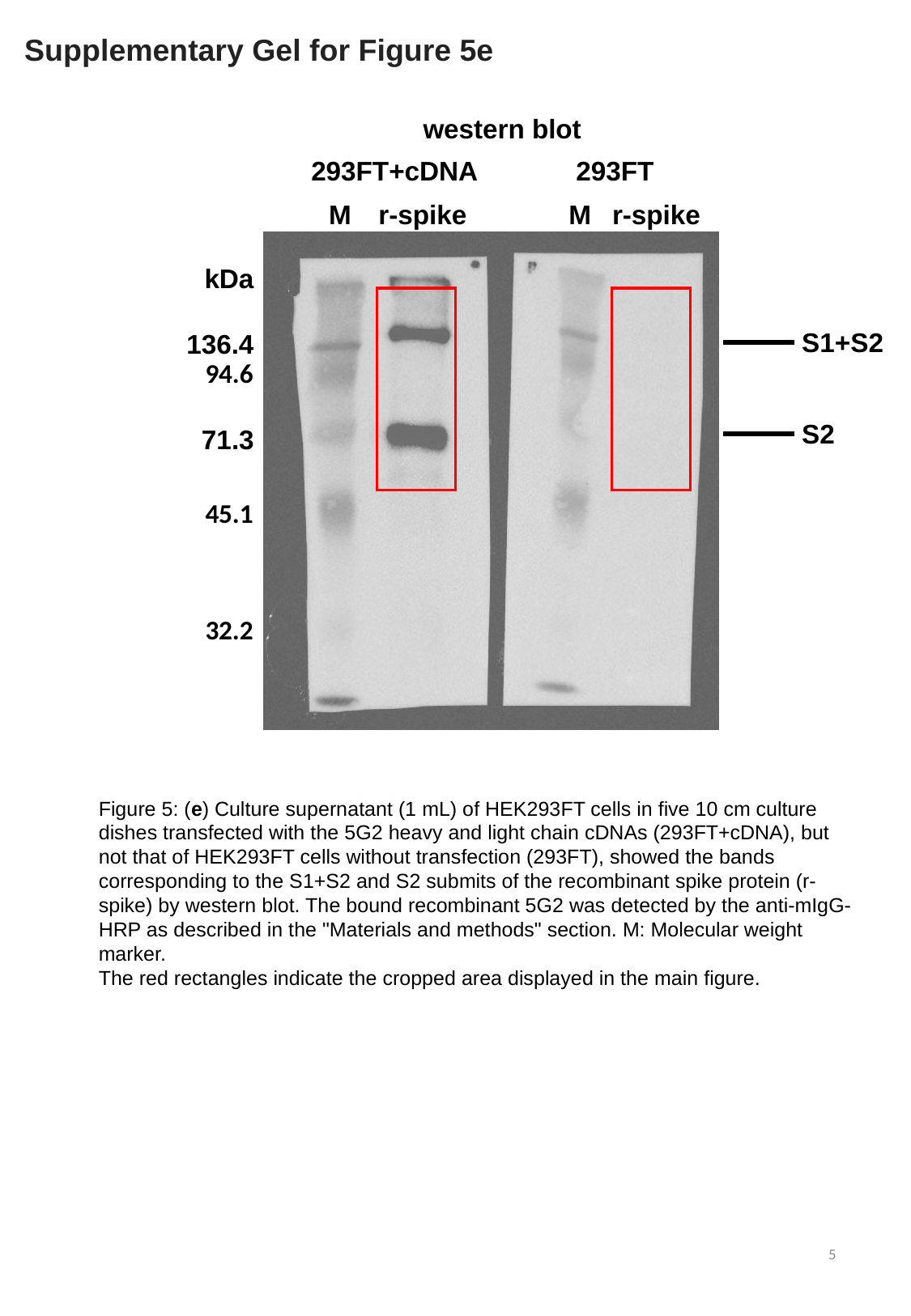

Supplementary Gel for Figure 5e
western blot
293FT+cDNA
293FT
M
r-spike
M
r-spike
kDa
S1+S2
136.4
94.6
S2
71.3
45.1
32.2
Figure 5: (e) Culture supernatant (1 mL) of HEK293FT cells in five 10 cm culture dishes transfected with the 5G2 heavy and light chain cDNAs (293FT+cDNA), but not that of HEK293FT cells without transfection (293FT), showed the bands corresponding to the S1+S2 and S2 submits of the recombinant spike protein (r-spike) by western blot. The bound recombinant 5G2 was detected by the anti-mIgG-HRP as described in the "Materials and methods" section. M: Molecular weight marker.
The red rectangles indicate the cropped area displayed in the main figure.
5

## Slide 6
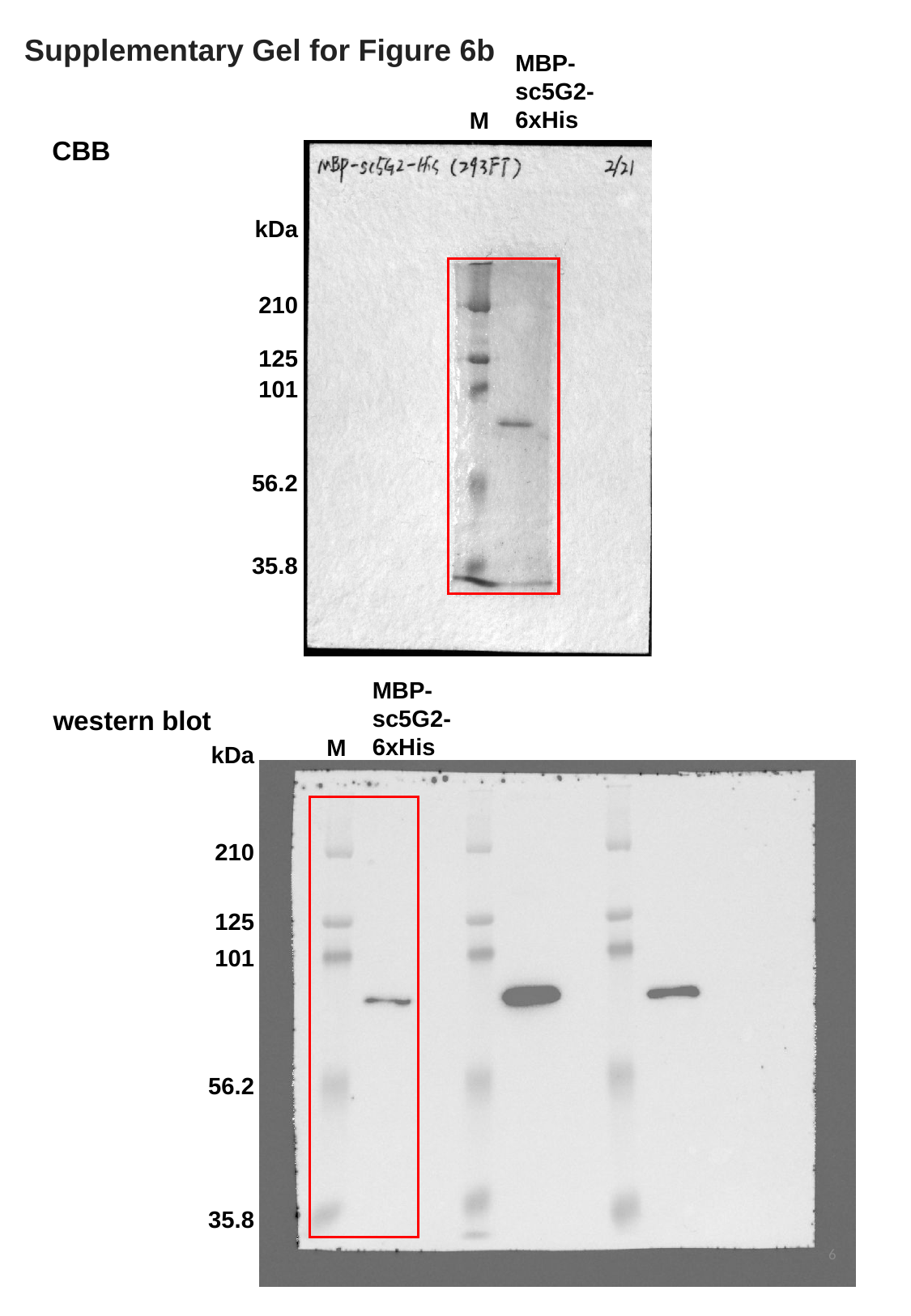

Supplementary Gel for Figure 6b
MBP-
sc5G2-
6xHis
M
CBB
kDa
210
125
101
56.2
35.8
MBP-
sc5G2-
6xHis
western blot
M
kDa
210
125
101
56.2
35.8
6

## Slide 7
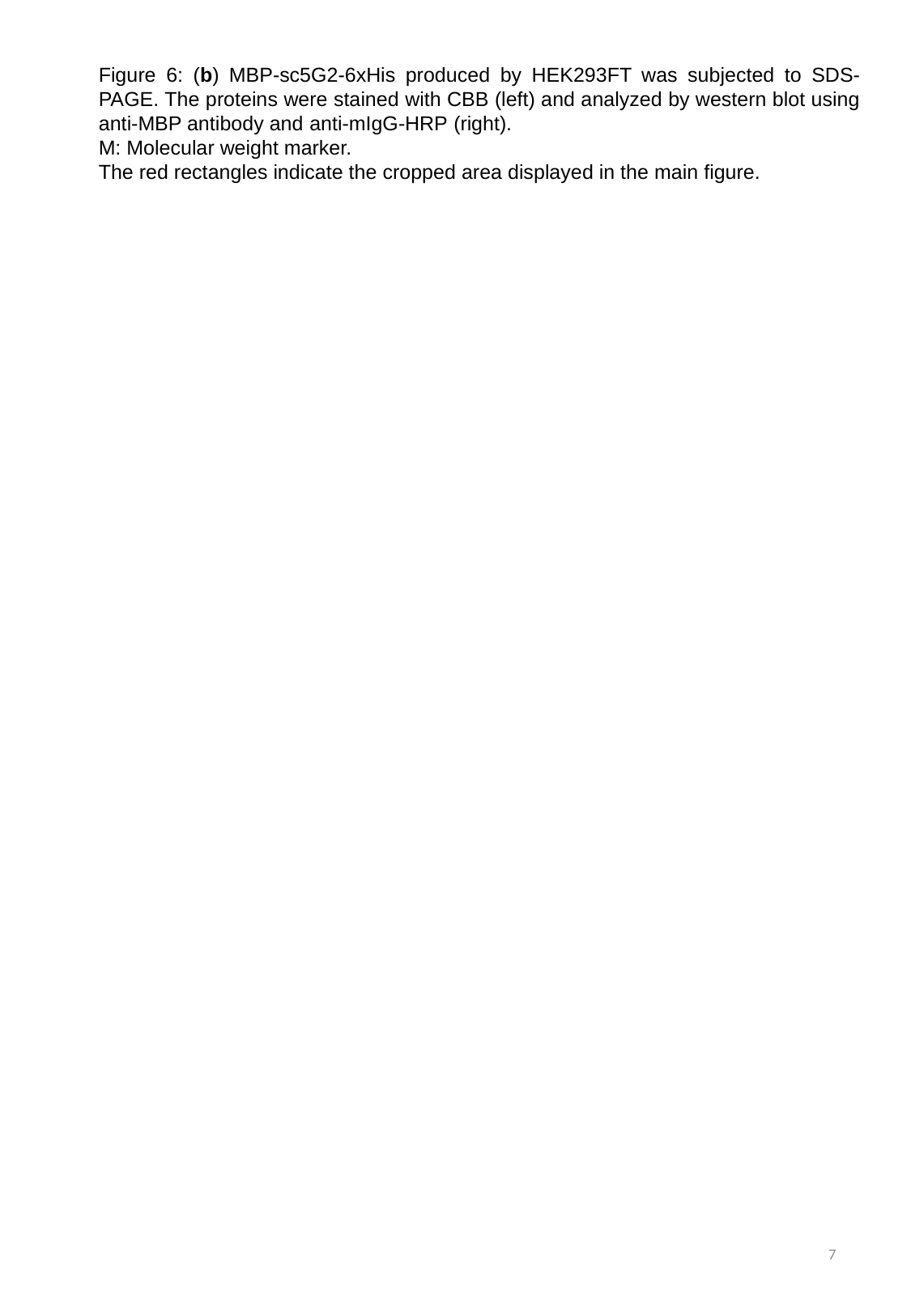

Figure 6: (b) MBP-sc5G2-6xHis produced by HEK293FT was subjected to SDS-PAGE. The proteins were stained with CBB (left) and analyzed by western blot using anti-MBP antibody and anti-mIgG-HRP (right).
M: Molecular weight marker.
The red rectangles indicate the cropped area displayed in the main figure.
7

## Slide 8
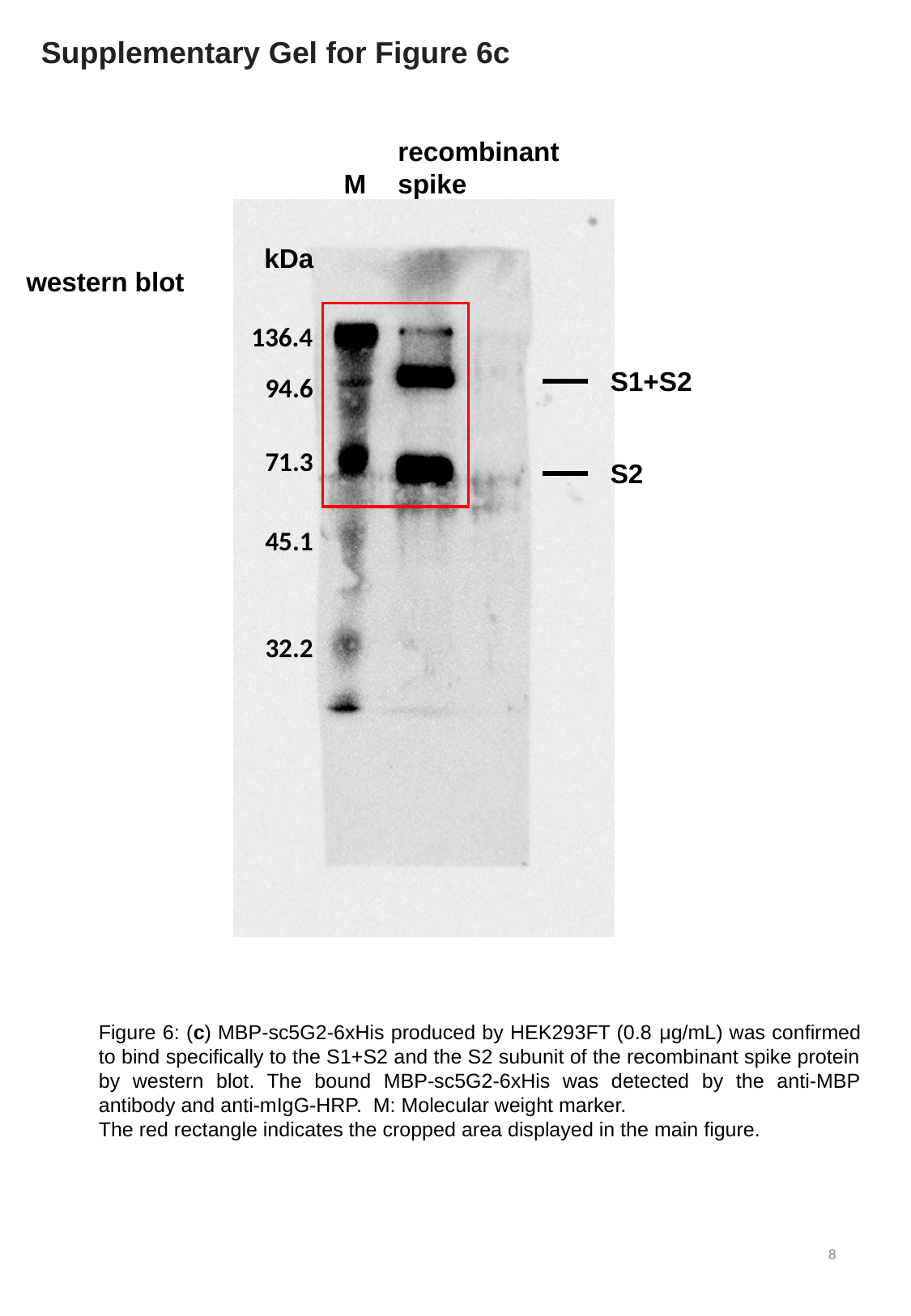

Supplementary Gel for Figure 6c
recombinant
spike
M
kDa
western blot
136.4
S1+S2
94.6
71.3
S2
45.1
32.2
Figure 6: (c) MBP-sc5G2-6xHis produced by HEK293FT (0.8 μg/mL) was confirmed to bind specifically to the S1+S2 and the S2 subunit of the recombinant spike protein by western blot. The bound MBP-sc5G2-6xHis was detected by the anti-MBP antibody and anti-mIgG-HRP. M: Molecular weight marker.
The red rectangle indicates the cropped area displayed in the main figure.
8

## Slide 9
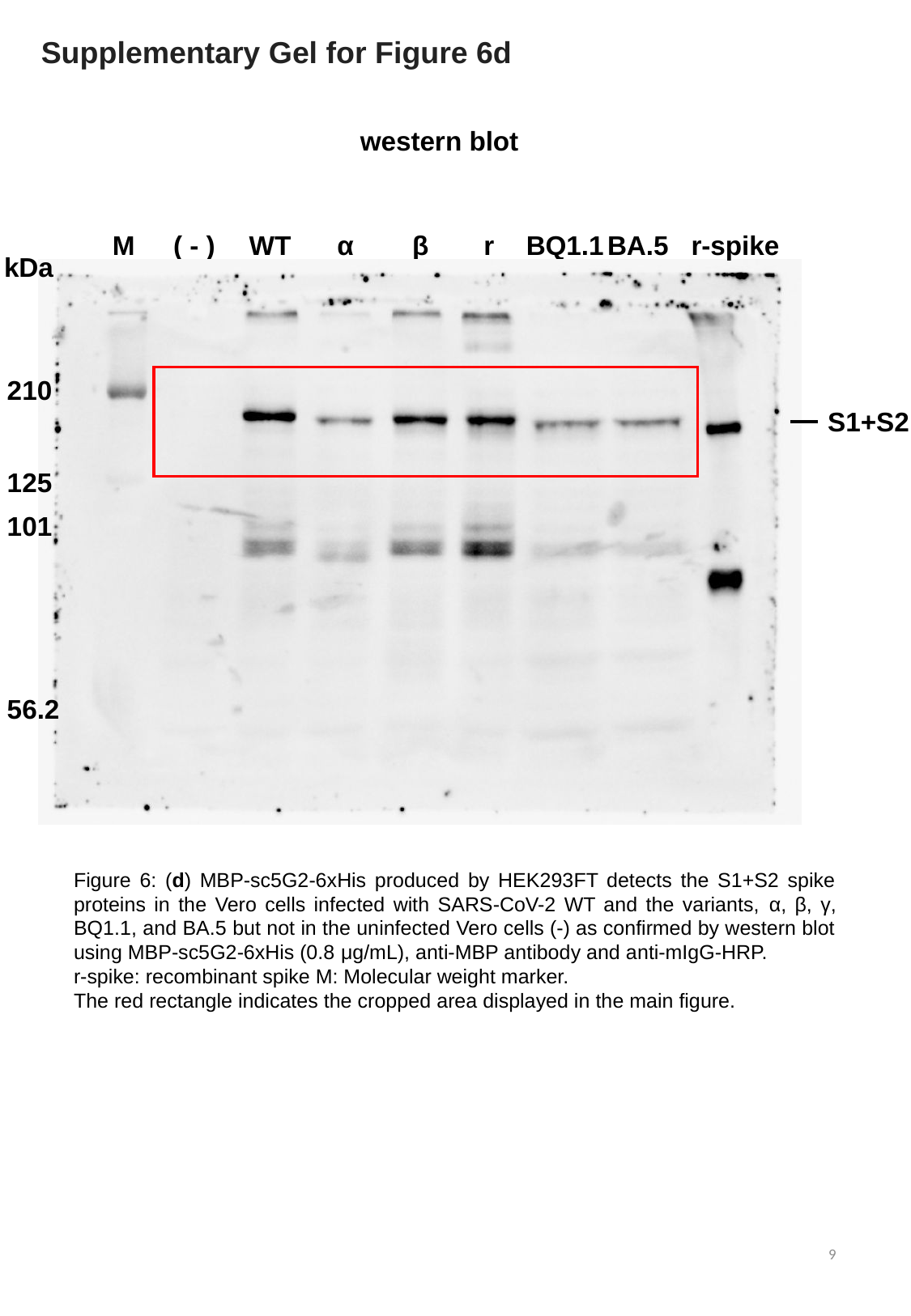

Supplementary Gel for Figure 6d
western blot
M
( - )
WT
α
β
r
BQ1.1
BA.5
r-spike
kDa
210
S1+S2
125
101
56.2
Figure 6: (d) MBP-sc5G2-6xHis produced by HEK293FT detects the S1+S2 spike proteins in the Vero cells infected with SARS-CoV-2 WT and the variants, α, β, γ, BQ1.1, and BA.5 but not in the uninfected Vero cells (-) as confirmed by western blot using MBP-sc5G2-6xHis (0.8 μg/mL), anti-MBP antibody and anti-mIgG-HRP.
r-spike: recombinant spike M: Molecular weight marker.
The red rectangle indicates the cropped area displayed in the main figure.
9

## Slide 10
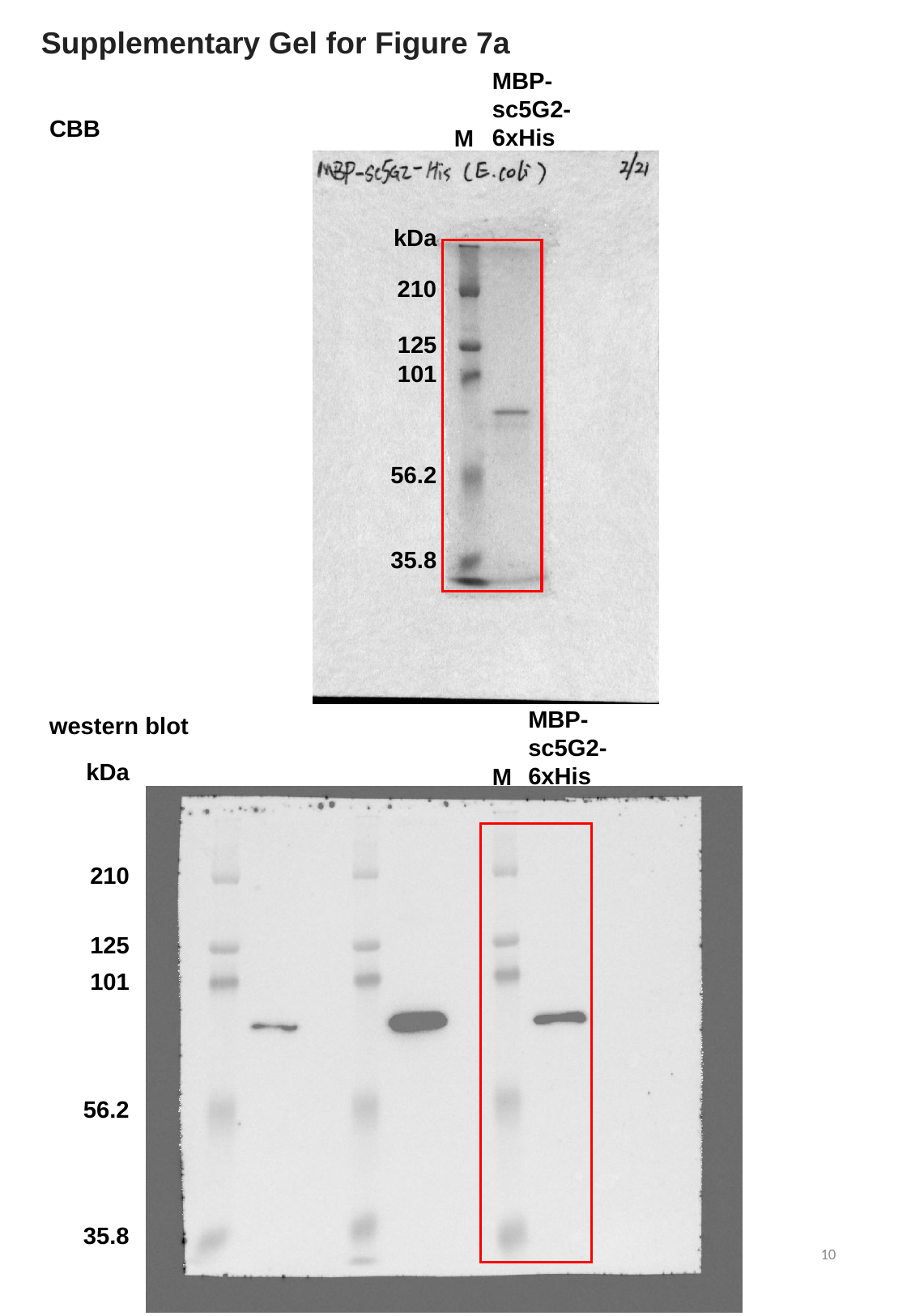

Supplementary Gel for Figure 7a
MBP-
sc5G2-6xHis
CBB
M
kDa
210
125
101
56.2
35.8
MBP-
sc5G2-6xHis
western blot
kDa
M
210
125
101
56.2
35.8
10

## Slide 11
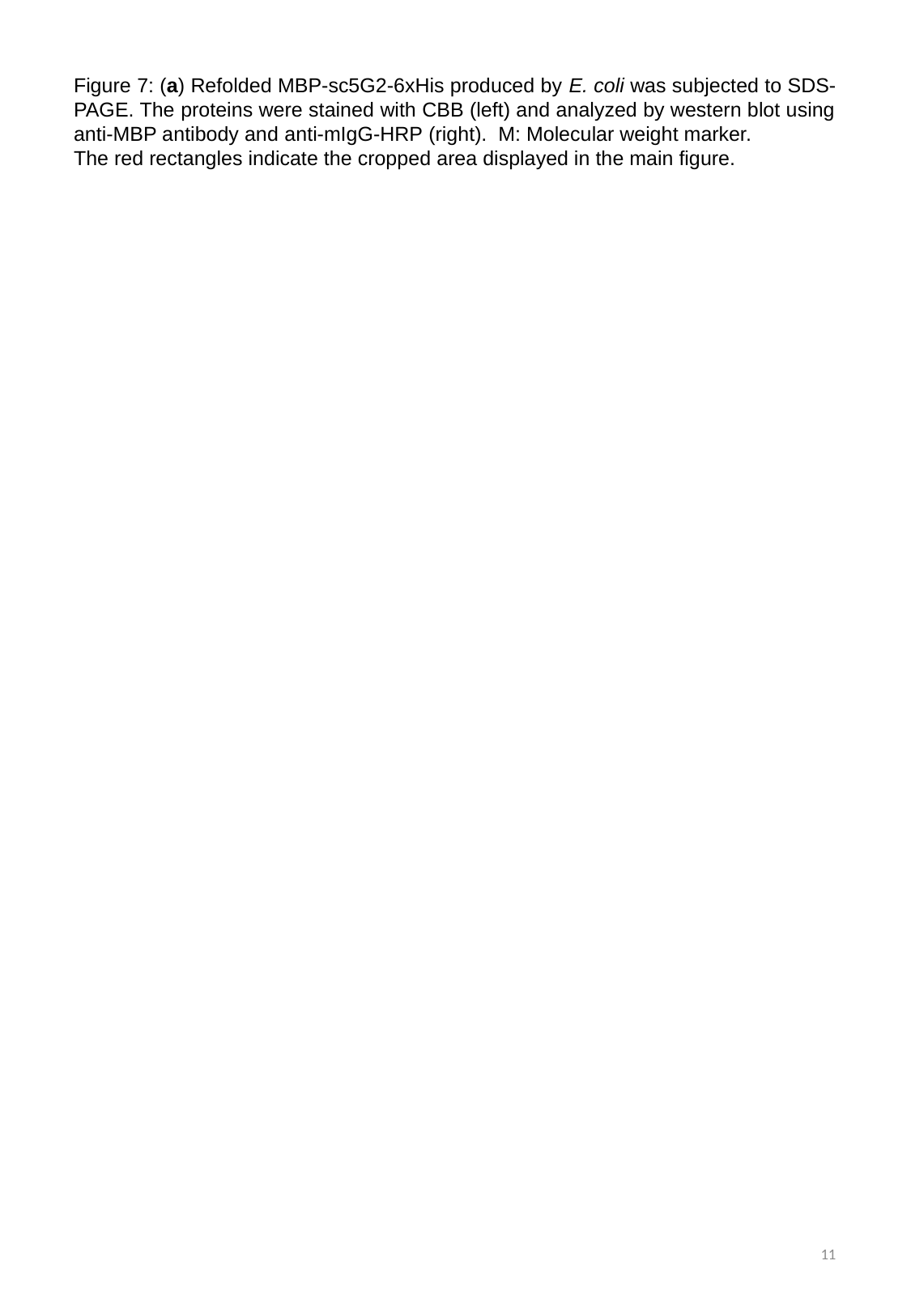

Figure 7: (a) Refolded MBP-sc5G2-6xHis produced by E. coli was subjected to SDS-PAGE. The proteins were stained with CBB (left) and analyzed by western blot using anti-MBP antibody and anti-mIgG-HRP (right). M: Molecular weight marker.
The red rectangles indicate the cropped area displayed in the main figure.
11

## Slide 12
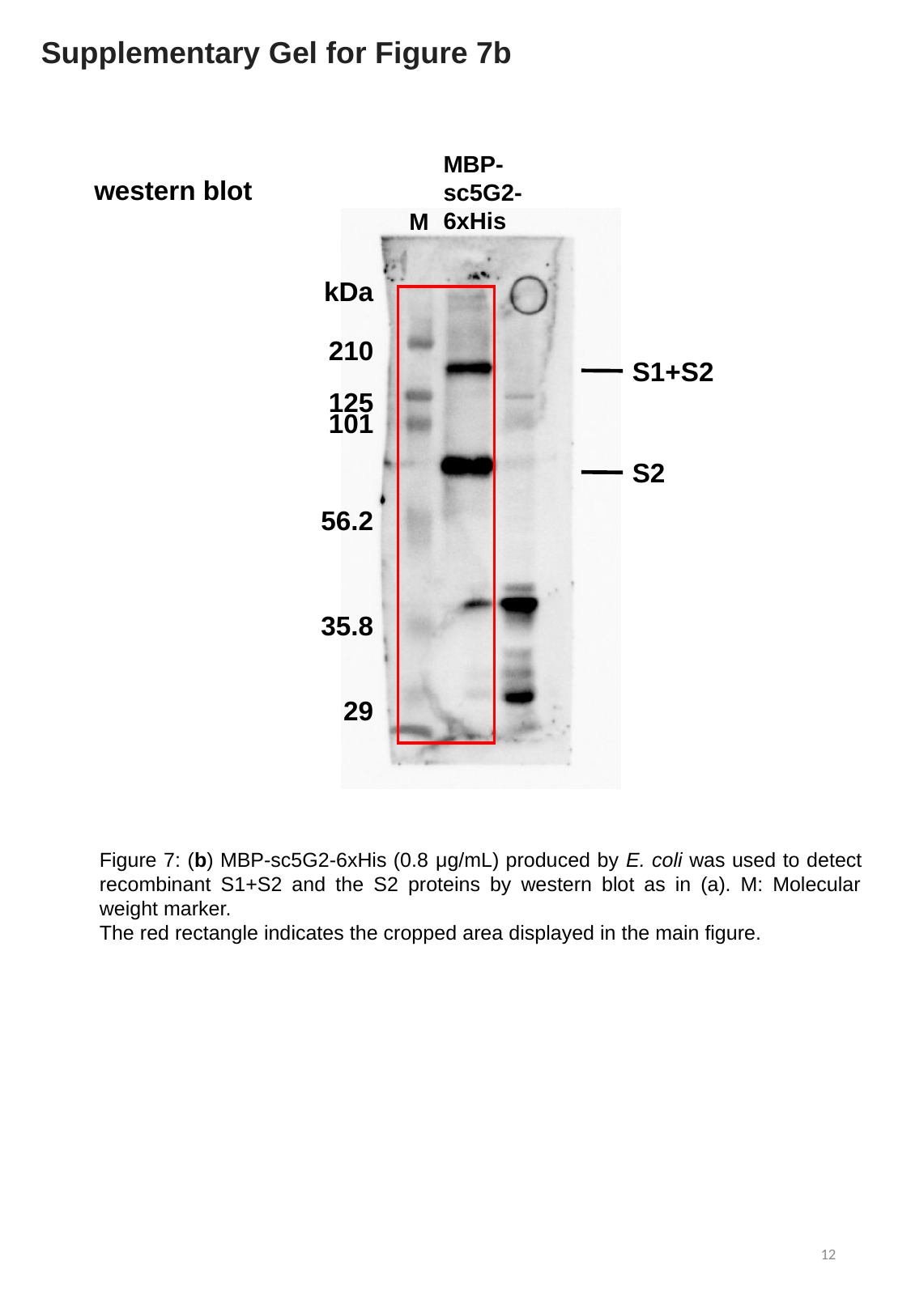

Supplementary Gel for Figure 7b
MBP-
sc5G2-6xHis
western blot
M
kDa
210
S1+S2
125
101
S2
56.2
35.8
29
Figure 7: (b) MBP-sc5G2-6xHis (0.8 μg/mL) produced by E. coli was used to detect recombinant S1+S2 and the S2 proteins by western blot as in (a). M: Molecular weight marker.
The red rectangle indicates the cropped area displayed in the main figure.
12

## Slide 13
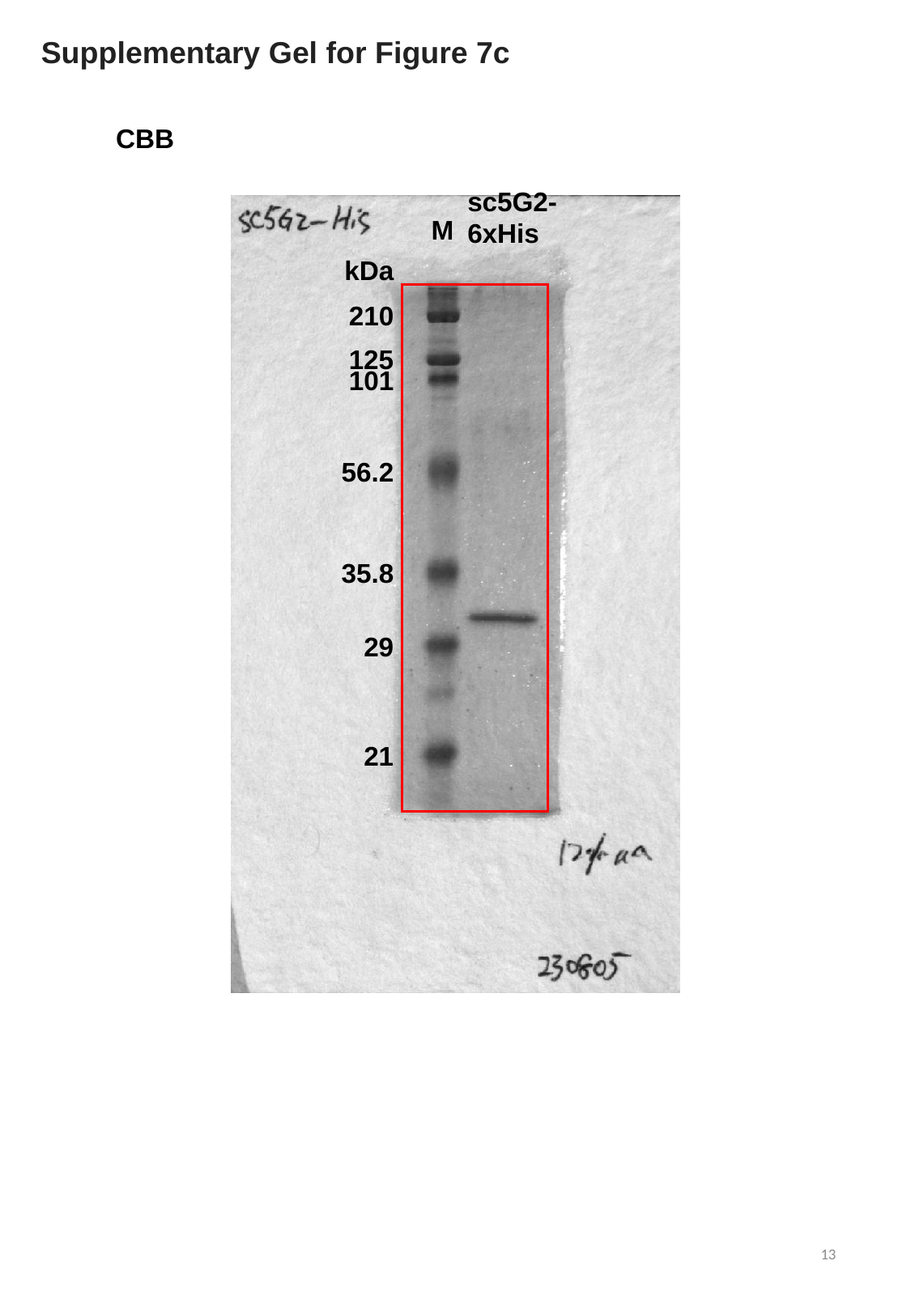

Supplementary Gel for Figure 7c
CBB
sc5G2-6xHis
M
kDa
210
125
101
56.2
35.8
29
21
13

## Slide 14
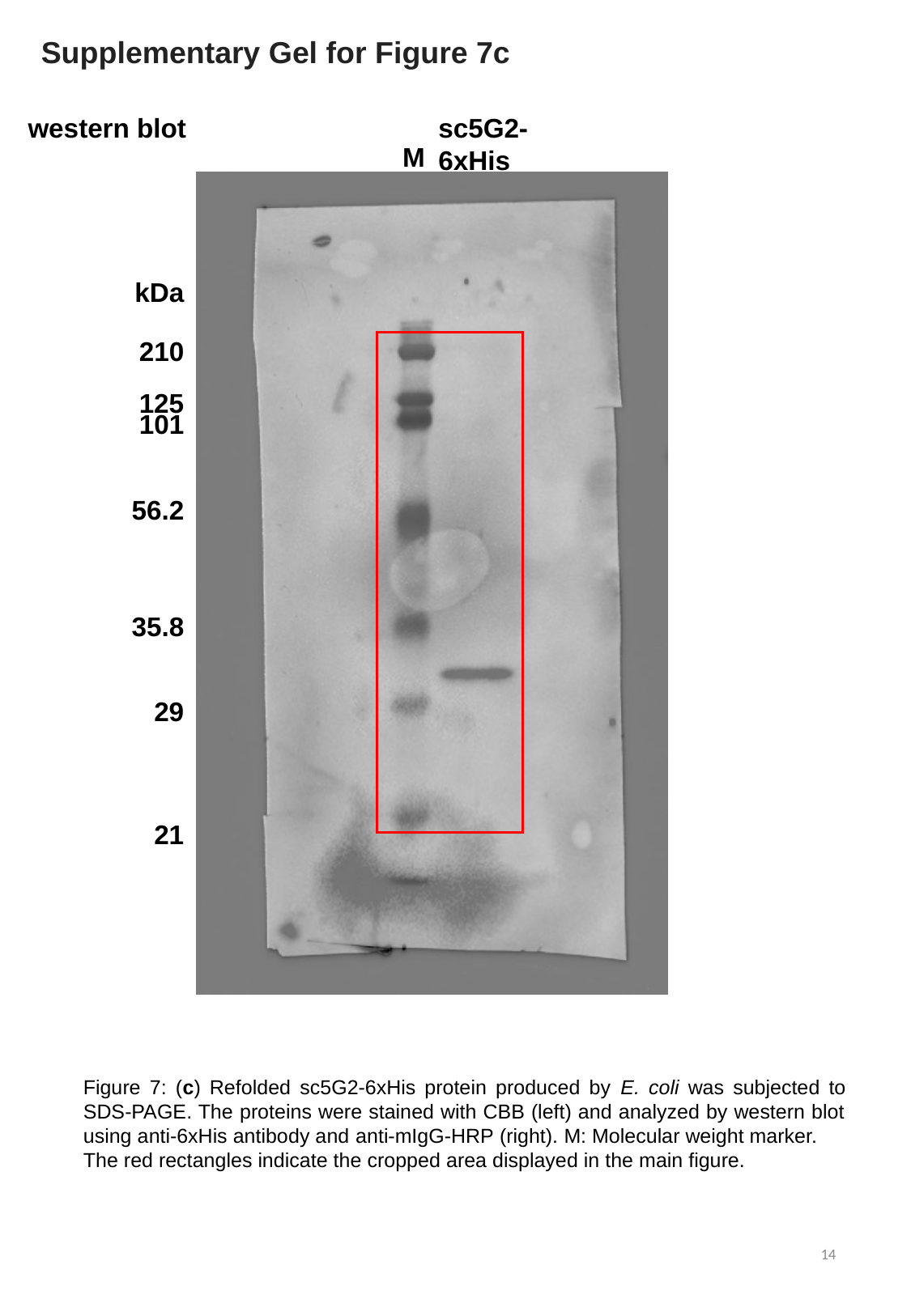

Supplementary Gel for Figure 7c
western blot
sc5G2-6xHis
M
kDa
210
125
101
56.2
35.8
29
21
Figure 7: (c) Refolded sc5G2-6xHis protein produced by E. coli was subjected to SDS-PAGE. The proteins were stained with CBB (left) and analyzed by western blot using anti-6xHis antibody and anti-mIgG-HRP (right). M: Molecular weight marker.
The red rectangles indicate the cropped area displayed in the main figure.
14

## Slide 15
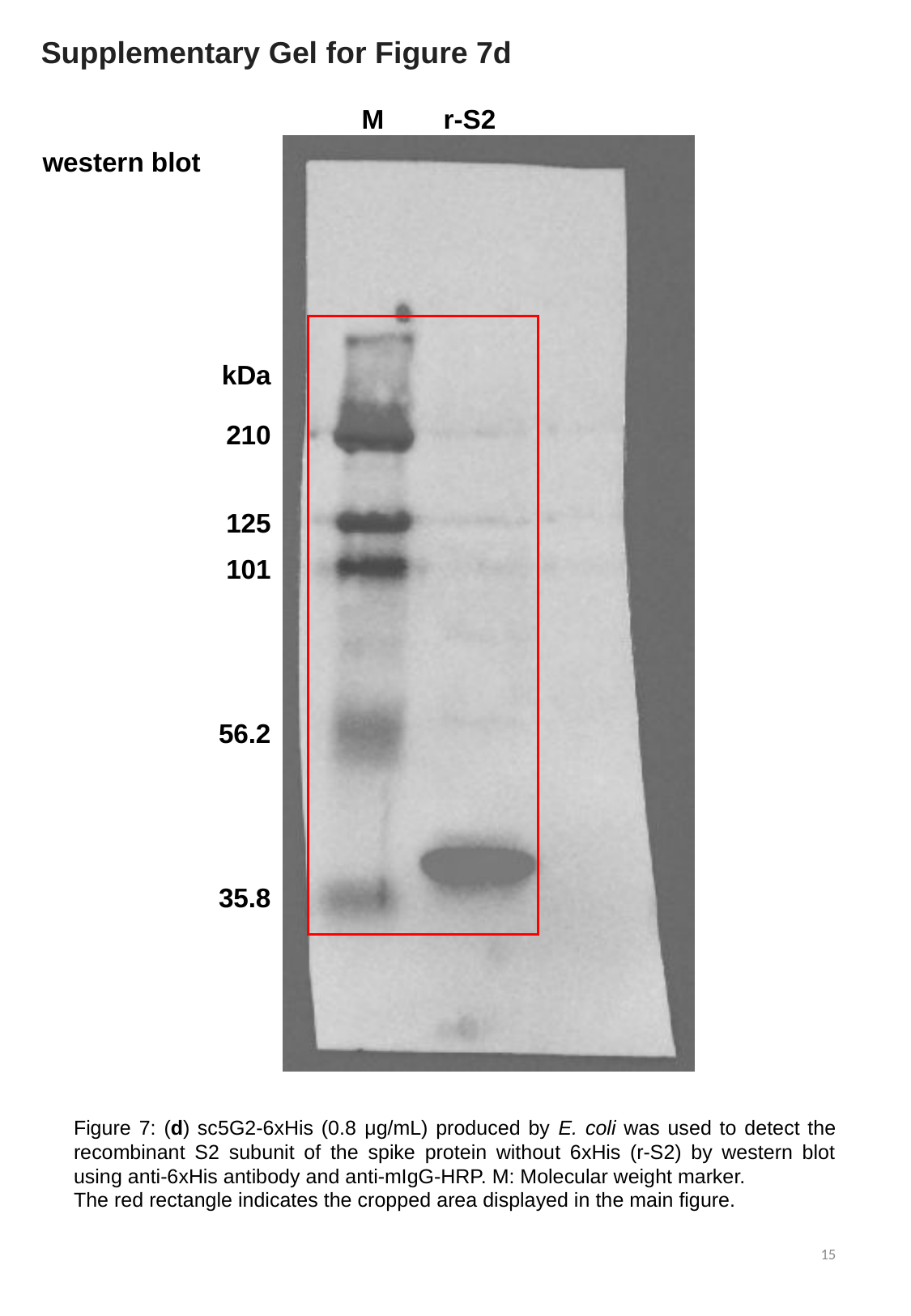

Supplementary Gel for Figure 7d
M
r-S2
western blot
kDa
210
125
101
56.2
35.8
Figure 7: (d) sc5G2-6xHis (0.8 μg/mL) produced by E. coli was used to detect the recombinant S2 subunit of the spike protein without 6xHis (r-S2) by western blot using anti-6xHis antibody and anti-mIgG-HRP. M: Molecular weight marker.
The red rectangle indicates the cropped area displayed in the main figure.
15

## Slide 16
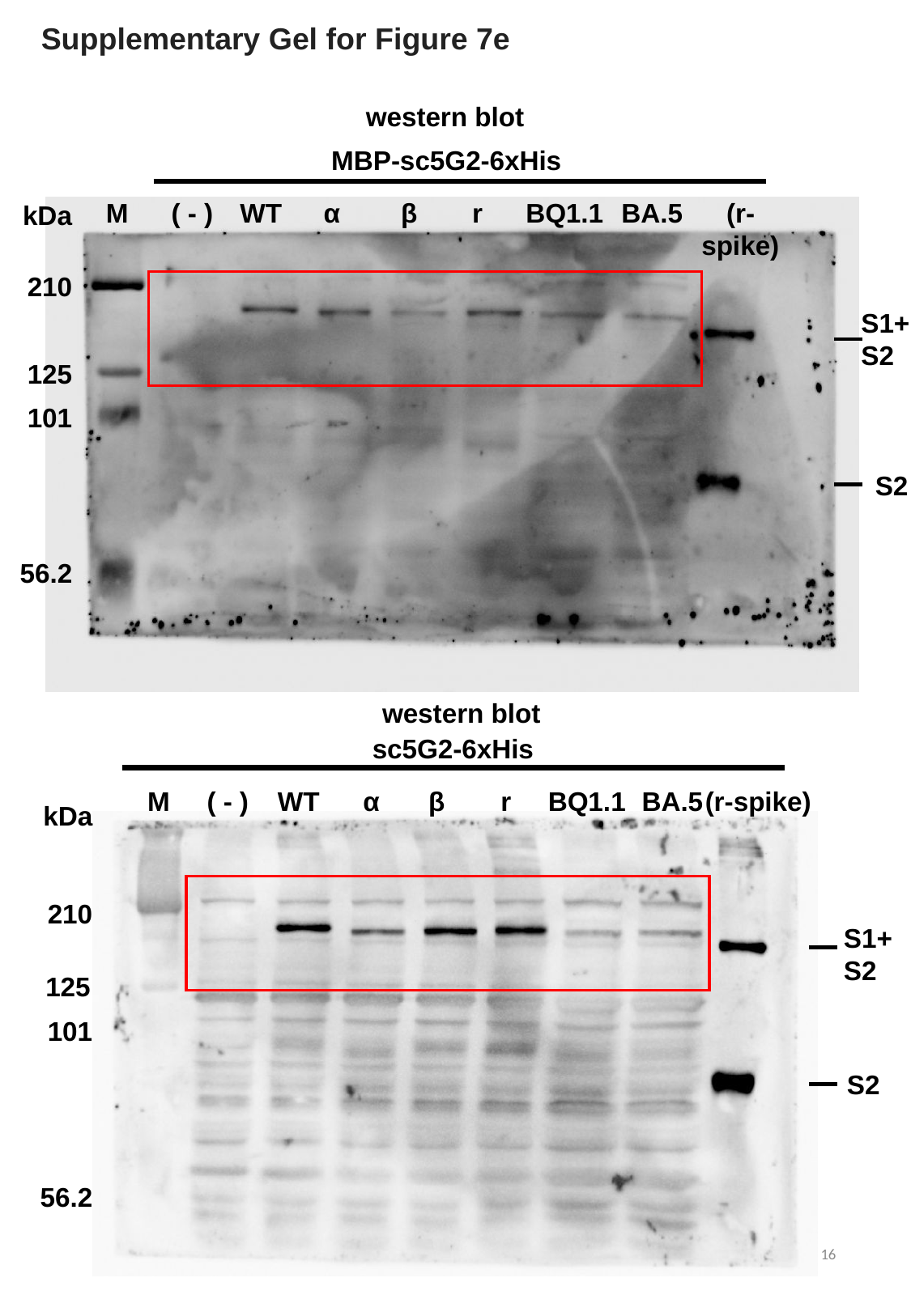

Supplementary Gel for Figure 7e
western blot
MBP-sc5G2-6xHis
M
( - )
WT
α
β
r
BQ1.1
BA.5
(r-spike)
kDa
210
S1+S2
125
101
S2
56.2
western blot
sc5G2-6xHis
M
( - )
WT
α
β
r
BQ1.1
BA.5
(r-spike)
kDa
210
S1+S2
125
101
S2
56.2
16

## Slide 17
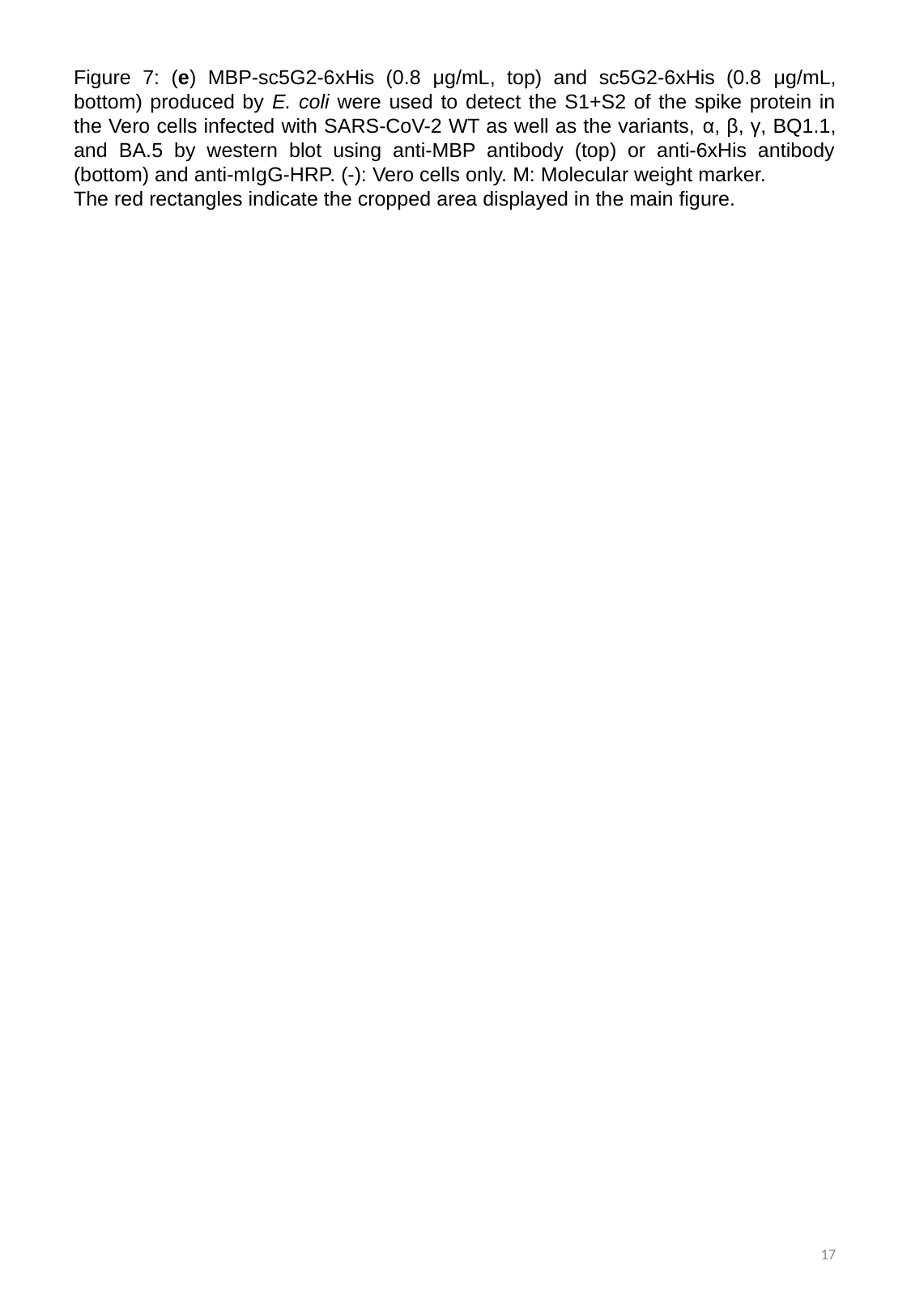

Figure 7: (e) MBP-sc5G2-6xHis (0.8 μg/mL, top) and sc5G2-6xHis (0.8 μg/mL, bottom) produced by E. coli were used to detect the S1+S2 of the spike protein in the Vero cells infected with SARS-CoV-2 WT as well as the variants, α, β, γ, BQ1.1, and BA.5 by western blot using anti-MBP antibody (top) or anti-6xHis antibody (bottom) and anti-mIgG-HRP. (-): Vero cells only. M: Molecular weight marker.
The red rectangles indicate the cropped area displayed in the main figure.
17
